# Immune Activation in the Female Genital Tract: Expression Profiles of Soluble Proteins in Women at High Risk for HIV Infection

**DOI:** 10.1371/journal.pone.0143109

**Published:** 2016-01-27

**Authors:** Suzanna C. Francis, Yanwen Hou, Kathy Baisley, Janneke van de Wijgert, Deborah Watson-Jones, Trong T. Ao, Carolina Herrera, Kaballa Maganja, Aura Andreasen, Saidi Kapiga, Gary R. Coulton, Richard J. Hayes, Robin J. Shattock

**Affiliations:** 1 MRC Tropical Epidemiology Group, London School of Hygiene and Tropical Medicine, London, United Kingdom; 2 Mwanza Intervention Trials Unit, National Institute for Medical Research, Mwanza, Tanzania, United Republic of Tanzania; 3 Division of Basic Medical Sciences, St. George's Medical School, University of London, London, United Kingdom; 4 Institute of Infection and Global Health, University of Liverpool, Liverpool, United Kingdom; 5 Mucosal Infection and Immunity Group, Imperial College, Department of Medicine, London, United Kingdom; 6 Clinical Research Department, London School of Hygiene and Tropical Medicine, London, United Kingdom; National Cancer Institute, UNITED STATES

## Abstract

Soluble cervicovaginal biomarkers of inflammation, immune activation and risk of HIV acquisition are needed to reliably assess the safety of new biomedical prevention strategies including vaccines and microbicides. However, a fuller understanding of expression profiles in women at high risk for HIV infection is crucial to the effective use of these potential biomarkers in Phase 3 trial settings. We have measured 45 soluble proteins and peptides in cervicovaginal lavage samples from 100 HIV negative women at high risk for HIV infection. Women were followed over one menstrual cycle to investigate modulation by hormonal contraception, menstrual cycle phase, recent sexual exposure and intravaginal practices. Women using injectable DMPA had increased concentration of several soluble proteins of the innate and adaptive immune system, including IL-1α, IL-1β, IL-2, MIP-1β, IP-10, IL-8, TGF-β, HBD4, IgA, IgG1, and IgG2. Women using combined oral contraceptives had a similar signature. There were differences in concentrations among samples from post-ovulation compared to pre-ovulation, notably increased immunoglobulins. Increased prostate-specific antigen, indicative of recent sexual exposure, was correlated with increased IL-6, MCP-1, and SLPI, and decreased GM-CSF and HBD3. The identified signature profiles may prove critical in evaluating the potential safety and impact on risk of HIV acquisition of different biomedical intervention strategies.

## Introduction

The HIV pandemic continues to expand, with an average of 2.5 million new infections per year [1]. The majority of these new infections are in sub-Saharan Africa, where the epidemic is driven by heterosexual transmission and women make up 60% of the epidemic [1]. Safe, effective, female-controlled HIV prevention methods are urgently needed. Although there have been recent successes with oral pre-exposure prophylaxis, a topical vaginal microbicide and a parenteral vaccine [2–7], these products have been only partially protective, and the search continues for more robust methods. Investigations following unsuccessful products in Phase 3 clinical trials, especially for products associated with increased rates of HIV infection such as nonoxynol-9 [8], cellulose sulphate [9], and recombinant Adenovirus-5 HIV vaccines [10], have highlighted the need to better understand immune activation in the female genital tract for early safety assessment.

Immune activation in the female genital tract can be caused by infection, irritation or epithelial trauma, and results in increased or decreased expression of soluble immune proteins [11], and has been shown to result in attraction of cells expressing HIV co-receptors to the cervicovaginal mucosa thereby increasing susceptibility to HIV infection [8]. Evidence from several trials of ineffective or harmful microbicides has shown that some candidate products can increase concentration of inflammatory immune proteins [8]. Increasingly, clinical studies are measuring soluble immune biomarkers to screen for product-induced mucosal toxicity/irritation in pre-clinical and clinical trials [12–17]. The most common soluble proteins evaluated in trials have been interleukin (IL)-1α, IL-1β, IL-1-receptor antagonist, IL-6, IL-8, tumour necrosis factor (TNF)-α and secretory leukocyte peptidase inhibitor (SLPI) [13–17]. Soluble immune biomarkers may also be useful for vaccine development; not only providing safety information for mucosal vaccines, but also for parenteral vaccines such as Adenovirus 5 that may increase immune activation at mucosal sites [18,19].

A number of biomedical and behavioural factors can influence expression of immune proteins in the female genital tract [12], and more research is needed to understand this background variation for future clinical trials. Two studies have investigated baseline variation in low risk populations appropriate for Phase I clinical trials [20,21]; however, only one study has investigated baseline variation among women at high risk for HIV infection in sub-Saharan Africa [22]. There is evidence that soluble protein concentrations vary by menstrual cycle phase [23,24], hormonal contraception use [20,25], seminal plasma exposure [26], the composition of the vaginal microbiota [22,27], and the presence of infections, including sexually transmitted infections (STIs) [28–30]. In sub-Saharan Africa, the effect of highly prevalent vaginal practices, such as intravaginal cleansing, on immune proteins has only been investigated in one study [22,31]. Lastly, many of the studies have focused on pro-inflammatory cytokines and chemokines and, to a lesser extent, growth factors and antimicrobial proteins or peptides. Investigating a wide array of soluble proteins in the female genital tract, including immunoglobulins, may be useful for improving our understanding of their interactions.

To address these gaps, we measured the levels of 45 different soluble immune and antimicrobial proteins and peptides in cervicovaginal lavages (CVL) from 100 women participating in an intensive longitudinal sub-study of a larger microbicide feasibility study in North-West Tanzania. Soluble analytes evaluated included pro-inflammatory cytokines, anti-inflammatory cytokines, growth factors, chemokines, antimicrobial proteins and immunoglobulins. We present data from visits without known STIs, and report on the concentrations of these analytes and the association with menstrual cycle, hormonal contraception, seminal plasma exposure, reported intravaginal practices and clinical findings.

## Materials and Methods

### Ethics Statement

All study procedures were approved by the ethics committees of the London School of Hygiene and Tropical Medicine and the Medical Research Coordinating Committee of the Tanzanian National Institute for Medical Research. All participants received detailed information about the study to ensure that they understood why the study was being carried out and what the study involved. Informed consent was obtained by signature if literate, or thumb-printed and witnessed (if illiterate) prior to their participation in the study.

### Study participants

This study was nested within a 12 month microbicide feasibility study of 970 HIV-negative women aged 18–44 years working in bars, hotels, and other food and recreational facilities in the three towns of Geita, Shinyanga and Kahama in northwestern Tanzania from 2008–2010. These towns are located near large-scale gold or diamond mines in which there are large populations of migratory male workers. This study showed a high HIV prevalence (18%) and incidence (3.7 cases per 100 woman-years) [32]. Similar populations have been targeted for recent HIV prevention trials in Tanzania due to high prevalence and incidence of HIV [33,34]. The purpose of the microbicide feasibility study was to assess feasibility, retention and appropriateness of this population for future trials, and screening, enrolment and follow-up procedures for this cohort have been described elsewhere [32]. We enrolled 100 women into a sub-study between August and October 2009. Women were sampled purposefully by reported intravaginal practices at cohort enrolment in order to ensure inclusion of women using less common intravaginal practices (e.g. insertion with detergents). Women who were HIV positive, pregnant, currently breastfeeding, or had previous known cervical or uterine abnormalities or surgery (“Have you ever been told that you have a problem with your cervix or uterus?” and “Have you ever had an operation on your cervix or uterus?”) were excluded from the study. Women who were menstruating during the enrolment visits were asked to return after the completion of menses.

### Study design

Participants enrolled in the sub-study were followed up three times a week for 4 weeks (12 visits total). At enrolment, interviews were carried out to obtain information about sexual behaviour, vaginal practices, current contraception, and STI symptoms. On the first and last visit (visits 1 & 12), a clinical and colposcopic examination were performed; cervical and vaginal swabs were collected to test for vaginal pH and reproductive tract infections (*Chlamydia trachomatis*, *Neisseria gonorrhoeae*, *Trichomonas vaginalis*, bacterial vaginosis, and yeast); and a CVL was obtained for the detection of *Herpes simplex virus*, *types 1 and 2* (HSV), prostate-specific antigen (PSA) to measure seminal plasma exposure in the last 48 hours [35], soluble immune proteins, haemoglobin and white blood cells (WBCs). During visits 2 to 11, a shortened interview was conducted to obtain updated sexual behaviours and intravaginal practices. A brief clinical examination was performed to obtain vaginal swabs for testing vaginal pH, bacterial vaginosis and yeast, and a CVL for the detection of HSV, PSA, soluble immune proteins, haemoglobin and WBCs. Urine was collected at every visit to test for pregnancy and menstrual cycle phase. If a woman was menstruating, no genital samples were obtained on that visit. No blood samples were collected; however laboratory data from the main cohort on HSV antibody status, HIV status and syphilis results were available for the statistical analysis and methods have been reported in a previous study [32]. Colposcopy was carried out by trained clinicians according to the CONRAD/WHO revised manual for the Standardization of Colposcopy for the Evaluation of Vaginal Products [36].

### Sample processing and laboratory testing

#### CVL sample processing

CVL samples were obtained by flushing 5ml of sterile normal saline with a sterile pipette over the cervix and the lateral vaginal walls. After 60 seconds, the fluid was aspirated from the posterior fornix using the same pipette and collected in a 15 ml conical polypropylene tube that was then stored temporarily in a cool box with ice (2–8°C) before processing. Visual appearance was noted and documented, and 10μl of samples was used to assess the sample for haemoglobin using Hemastix® reagent indicator strips (Bayer Diagnostics, Tarrytown, NY, USA), comparing the indicator colouring with colour categories representing approximate quantities of erythrocytes (ery) per uL as specified by the manufacturer: none, low (25 ery/μL), moderate (80 ery/μL), high (200 ery/μL). CVLs were centrifuged onsite at 3500 rpm for 10 minutes within two hours of collection. Protease inhibitor (Cocktail Set I, Calbiochem, Merck Millipore, Darmstadt, Germany) was added to two 1 ml aliquots for immune protein testing, and two 1ml aliquots were stored for batch testing for PSA and HSV viral quantification. CVL supernatant aliquots were stored at -20C° for 3 days and then at -80°C for long-term storage before shipping or batch testing.

We developed a method to enumerate the WBCs in the cell pellet modified from a manual blood count: the cell pellet was spread across a glass slide, fixed with methanol and air-dried. Leishman’s stain was applied to the slide for 10 minutes. With a light microscope at 100 times magnification, WBCs were counted and recorded on a differential cell counter until 100 WBCs were counted. Erythrocytes and epithelial cells were enumerated on a separate counter, and were not included in the 100-cell differential count.

#### Measurement of vaginal pH and reproductive tract infections

At the first and last visit, an endocervical swab was tested for detection of *N*. *gonorrhoeae* and *C*. *trachomatis* by Amplicor PCR kits (Roche Diagnostics, Branchburg, USA). All positive tests for *N*. *gonorrhoeae* were confirmed using specific primers to the 16S DNA coding region in PCR in-house assays [37]. A vaginal swab was obtained to inoculate a culture (TV InPouch, Biomed Diagnostics, San Jose, USA), which was read for the presence of motile trichomonads by light microscopy at 72 hours after incubation at 36–37°C.

At each visit, CVL supernatants were tested for HSV shedding using Artus HSV-1/2 PCR kits (Qiagen, Hilden, Germany). A vaginal swab was Gram-stained and examined and Nugent scored for diagnosis of bacterial vaginosis [38]. A third swab was rolled onto a pH indicator strip (ThermoFisher, Waltham, MA, USA); the indicator colouring was compared with colour zones representing the following pH values: 3.6; 4.1; 4.4; 4.7; 5.0; 5.3; 5.6 and 6.1. The same swab was used for wet mount microscopy for the detection of yeast hyphae or buds.

#### Measurement of PSA

We measured PSA at every visit using a quantitative PSA ELISA Kit (Calbiotech, Inc, Spring Valley, CA, USA) for the detection of PSA in human serum for cancer detection. A random selection of CVL supernatant aliquots were shipped to the Institute of Tropical Medicine in Antwerp, Belgium for evaluation against the SERATEC PSA Semiquant (Göttingen, Germany). The SERATEC test is a semi-quantitative chromatographic immunoassay also originally developed for human serum for cancer detection, and subsequently validated for the detection of PSA in vaginal fluid for professional forensic purposes with a sensitivity and specificity of 100% [39]. The Calbiotech test was found to be 70% sensitive and 100% specific for PSA in the cervical vaginal lavages against SERATEC when combining low positive (<4ng/ml) and negative values.

#### Measurement of immune proteins and peptides

Twenty-three soluble immune protein were quantified by in house multiplex bead immunoassay as previously described (Panels a and b of Figure A in S1 Fig) [40,41]. Following the same protocols, two more panels were added to measure human beta defensins(HBD)3, apolipoprotein(APO)A1, squamous cell carcinoma antigen(SCCA)-1, polymeric immunoglobulin receptor (PIGR), IL-10, IL-17, IL-18 and transforming growth factor(TGF)-α (Panel c of Figure A in S1 Fig) and SLPI, elafin, HBD2, HBD3, α-defensin/human neutrophil peptide (HNP) 1–3, involucrin and S100 calcium binding protein A8 (S100a8) (Panel e of Figure A in S1 Fig). Additionally, six immunoglobulin (Ig) isotypes (IgG1, IgG2, IgG3, IgG4, IgA, IgM) were measured using Milliplex map kit (Merck Millipore, Billerica, USA; Cat No: HGAM-301) following the manufacturer’s protocol (Panel d of Figure A in S1 Fig). Total protein was measured using Quick Start™ Bradford Protein Assay (Bio-Rad Laboratories, Hercules, USA).

#### Measurement of menstrual cycle phase

Urine aliquots were stored at -80°C for testing for Pregnanediol-3-Glucuronide (PDG, EIA, Immunometrics UK Ltd, London, United Kingdom), the principal metabolite of progesterone, and for testing of creatinine (CRT, R&D Systems, Inc., Minneapolis, MN, USA). Each PDG concentration was indexed by the CRT concentration of the same sample to adjust for urine dilution. We used a modified Kassam method to identify ovulatory cycles during the study; we used the minimum PDG/CRT value for each women during the study as the denominator, and the per-visit PDG/CRT value as the numerator [42]. A ratio threshold of greater than 4.0 signalled that ovulation had taken place [42,43]. This method has been used for twice weekly and every other day sampling, and found to be 100% sensitive and 77% specific with 6% misclassification [43]. In those women who reported no hormonal contraceptive use, who showed evidence of ovulation, and who had one observed menstruation, we then examined the PDG curves to identify the rise in PDG. We defined “Day 1” of the menstrual cycle as the first visit with observed menstruation. All visits after Day 1 were defined as pre-ovulation until a rise in PDG was seen, and all subsequent visits were defined as post-ovulation until menstruation. If there was no obvious PDG rise, visits were not categorized for analysis.

### Statistical analysis

Data were analyzed using Stata, version 12 (StataCorp, College Station, USA). We restricted the data set to “healthy visits” defined as visits without a positive STI test or reproductive tract infection. Visits were excluded from this analysis if results were positive for HSV shedding, vaginal yeast or bacterial vaginosis. In addition, all follow-up visits for women who tested positive at the first or last visit for *C*. *trachomatis*, *N*. *gonorrhoeae*, and *T*. *vagina*lis were excluded from this analysis.

CVL samples with analyte concentrations below the lower limit of quantification (LLOQ) of the assay were assigned a concentration of half the LLOQ. Those above the upper limit of quantification (ULOQ) were assigned values twice the ULOQ. Spearman’s rank correlation coefficient was calculated for each of the analytes within a Luminex panel to look for evidence of cross-reactivity (Figure A in S1 Fig).

The proportion of samples with concentration above the LLOQ, and the median and range of concentrations above the LLOQ, were calculated for each analyte. Since most analytes showed skewed distribution, concentrations were log10 transformed. To characterize the variation of the analytes over time between and within women, we used mixed-effects linear regression to estimate the ICC of each log-transformed value; variance components were estimated using residual maximum likelihood. The ICC was calculated as σ_B_^2^/ (σ_B_^2^ + σ_W_^2^), where σ_B_^2^ is the between-women variance and σ_W_^2^ is the within-woman variance. An ICC of 0 implies that observations from the same woman are no more similar to each other than they are to observations from different women. An ICC of 1 implies that all observations from the same woman are identical, so that the variation is due to between-woman differences. We also explored the effects of the dilution factor of the CVL by ICC (Table A in S1 Table and S1 Text).

We examined the association of analytes concentrations with the following exposures: menstrual cycle stage, hormonal contraception (reported use of combined oral contraceptive [COC] or injectable depot medroxyprogesterone acetate [DMPA]), seminal plasma, reported vaginal practices, cervical ectopy, colposcopic findings, and vaginal pH. Since detailed clinical examinations were only done at Visits 1 and 12, ectopy and colposcopy results were not available at other time points. To explore the association with the exposures of interest, we used mixed-effect linear regression for analytes with concentration >85% above LLOQ, or logistic regression with random effects for analytes with concentration ≤85% above LLOQ.

For the analysis, PSA was categorised into three levels: no PSA detected; low positive (<4ng/ml); and high positive (≥4ng/ml). Neutrophil counts were categorised into the following levels per 100 WBCs: no cells, 1–10 cells, 11–50 cells, >50 cells, and lymphocytes were analysed as presence/ absence. Vaginal pH results were categorised into the following levels: 3.6–4.1 (normal pH); 4.4–4.7 (high normal pH); and 5.0 and above (abnormal pH).

For each of the analyses of intravaginal cleansing with soap, intravaginal cleansing with cloth, and intravaginal insertion (i.e. the insertion of pulverized herbs or detergents), we compared samples from visits in which women reported the specific intravaginal practice to visits in which no cleansing or cleansing with fingers and water only was reported. We excluded visits in which women reported insertion of prescribed medications (e.g. treatment for candidiasis), leaving all but two women inserting detergents.

In the multivariable analysis, we considered age, reported sex in the past 3 days, and the presence of haemoglobin in the CVL as *a priori* confounders based on a conceptual model (Figure B in S1 Fig). For menstrual cycle phase, hormonal contraceptive use, intravaginal practices, clinical cervical ectopy, colposcopic abnormalities, and vaginal pH we controlled for the effects of age, reported sexual intercourse in the past three days and presence of haemoglobin; for PSA we controlled for age and presence of haemoglobin.

## Results

### Demographics, behavioural characteristics and clinical findings of the participants

One hundred participants attended 1,108 (92%) of 1,200 possible visits over follow-up. Of the 1,108 visits, 956 (86%) were non-menstruating, and of these, 370 (39%) met the definition of healthy visits; 67 women contributed at least one visit to this analysis. The participant demographic, behavioural, contraception and menstrual cycle data, and clinical and laboratory findings by visit are presented in Table 1. The mean age was 26 years old (range 18–44 years). Approximately one-quarter (28%) reported current use of hormonal contraception (i.e. COC or DMPA) at enrolment, 81% reported sexual intercourse during the study period, and 100% reported practicing vaginal cleansing during the study period. For cleansing, 81% used soap, 21% used a cloth (versus fingers alone), and 39% reported insertion of a substance into the vagina (e.g. herbs) at least once during the study.

**Table 1:** Demographics, behaviour and clinical characteristics.

Demographic and behavioural characteristics (N = 67)	N(%)
Age (*Mean*, *range)*	26 (18–44)
Reported vaginal intercourse during the study	54 (81%)
Reported intravaginal cleansing during the study	
Any cleansing	67 (100%)
Use of soap^1^	54 (81%)
Use of cloth	14 (21%)
Reported intravaginal insertion during the study	
Any insertion	26 (39%)
Medication	6 (9%)
Detergent^1^	18 (27%)
Herbs	1 (2%)
Tobacco	1 (2%)
**Contraception at enrolment (N = 67)**	
No contraception	19 (28%)
Condom use only	23 (34%)
Depot medroxyprogesterone acetate (DMPA)	13 (19%)
Combined oral contraceptive (COC)	6 (9%)
Sterilization	3 (5%)
IUD	1 (2%)
Other	2 (3%)
**Menstrual cycle**	
The number of cycles that could be assigned as ovulatory^2^ (n = 48)	46 (96%)
The number of menstruations during the study per participant^3^ (n = 46)	
No menstruation observed	11 (24%)
One menstruation	33 (72%)
Two menstruations	2 (4%)
Visits that could be assigned a menstrual cycle phase^4^ (n = 102 visits)	
Pre-ovulation	56 (55%)
Post-ovulation	46 (45%)
**Clinical and laboratory findings by visit (N = 370)**	
Cervical ectopy at V1 & V12 (n = 71 visits)	
Absent	43 (61%)
<20%	27 (38%)
≥20%	1 (1%)
Colposcopic findings^5^ at V1 & V12 (n = 71 visits)	
Absent	55 (78%)
Vaginal	0 (0%)
Fornices	1 (0.01%)
Cervical	15 (21%)
Vaginal pH measured at each visit^6^ (V1-V12; n = 361 visits)	
3.6–4.1	66 (19%)
4.4–4.7	145 (40%)
5.0 +	150 (72%)
Prostate specific antigen (PSA) measured at each visit (V1-V12; n = 370 visits)	
Negative	189 (51%)
Low positive (<4ng/ml)	118 (32%)
High positive (≥4ng/ml)	63 (17%)
Haemoglobin presence in CVL measured at each visit^7^ (V1-V12; n = 369 visits)	
Negative	123 (33%)
25 Ery/uL (+)	121 (33%)
80 Ery/uL (++)	60 (16%)
200 Ery/uL (+++)	65 (18%)

1. Soaps were defined as soaps meant for cleansing the body, while detergents were for cleaning clothes, dishes, etc.

2. We used a modified Kassam method to identify ovulatory cycles during the study; we used the minimum pregnanediol 3-glucuronide/ creatinine (PDG/CRT) value for each women during the study as the denominator, and the per-visit PDG/CRT value as the numerator. A ratio threshold of greater than 4.0 signalled that ovulation had taken place [51,52].

3. In those women who reported no hormonal contraceptive use and showed evidence of ovulation.

4. In those women who reported no hormonal contraceptive use, showed evidence of ovulation, and with one observed menstruation, pre and post ovulation was defined by the rise in pregnanediol 3-glucuronide.

5. The colposcopic findings on the fornix were petechiae (1 case), and on the cervix were petechiae (13 cases) erythema (1 case) and ecchymosis (1 case). There were no findings of oedema, grossly white findings, peeling, ulcer, abrasion or laceration.

6. Vaginal pH was measured using ThermoFisher pH test strips (range 3.6–6.1) with a colour chart.

7. Haemoglobin was measured using Hemastix® test strips with a colour chart.

There was evidence of ectopy at 28 of 71 (39%) possible healthy visits; the majority of cases of ectopy involved less than 20% of the cervix face. Colposcopy findings were detected at 21% (16/71) of visits: 15 cases were cervical findings (13 petechiae, 1 erythema and 1 ecchymosis); and one was on the anterior fornix (petechiae). There were no findings of oedema, grossly white findings, peeling, ulcer, abrasion or laceration.

In over half of the visits (58%), the vaginal pH was between 4.7 and 5.0, with only 14% of visits having a pH >5.0. Overall, 49% tested positive for PSA. Two-thirds of the samples (67%) tested positive for haemoglobin; 20% of samples with detectable haemoglobin were at visits flanking an observed menstrual period. However, of the 65 samples with high haemoglobin (≥200 ery/uL), only 46% were at the visit before and after an observed menstrual period. Thus, menstrual blood may have accounted for some, but not all of the haemoglobin found in the samples. WBCs were found in 51% of the CVL cell pellets, and these were mostly neutrophils with a lower proportion of lymphocytes.

In the 48 women who did not report hormonal contraception use, 46 (96%) showed signs of having ovulatory cycles; however, of these, 11 (24%) did not have an observed menstruation during the study period, and two had two separate menstruations. In the 33 women who showed evidence of ovulation and had one observed menstruation, 102 of 169 (63%) visits could be assigned as either pre-ovulatory or post-ovulatory.

### Expression patterns for soluble immune proteins in cervicovaginal lavages (CVLs)

**Fig 1:**
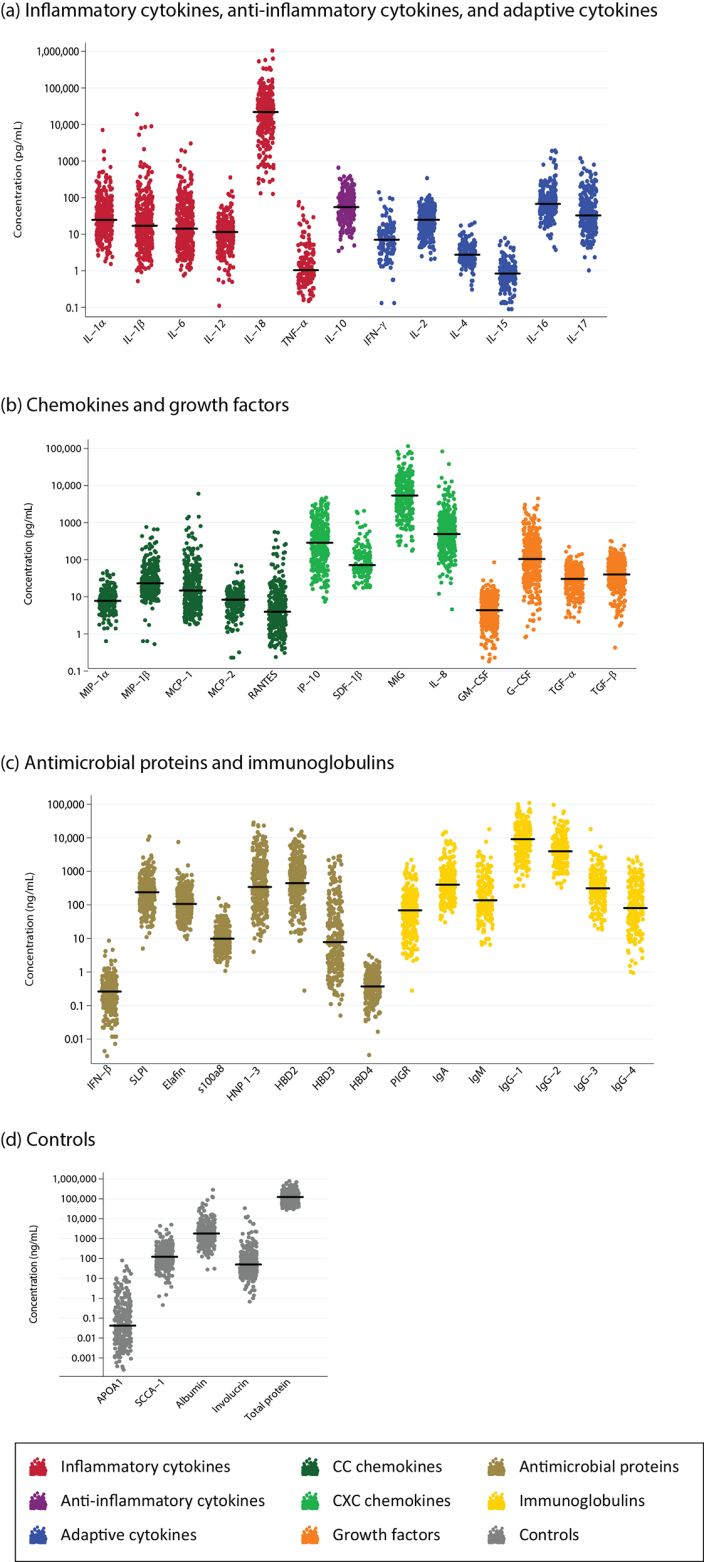
Distribution of analyte concentrations in cervicovaginal lavage samples for 370 healthy visits. Each data point represents a single sample and the line through data points represents the median concentration. APOA1 and albumin are serum controls and SCCA-1 and involucrin are vaginal epithelial controls.

Distributions of the analyte concentrations are visualized in Fig 1. Most analytes were detectable by the assays that were used, with only 13 out of 38 analytes having concentrations below LLOQ in ≥15% of visits at which they were measured (Table 2). For the comparison of biomedical and behavioural factors, these analytes were analysed as binary variables (i.e. presence/absence). Details of the median, range, mean and standard deviation, as well as percentages detected for each analyte from healthy visits are given in Table 2.

**Table 2:** The proportion of samples with analytes detected, median, mean and intra–class correlation coefficients (ICC) from 370 CVL samples.

Analyte	Percent with concentrations above LLOQ^1^	Median (range) CVL concentration	Mean (standard deviation) CVL concentration	σ; ICC; σ_w_ of log–transformed data^2^ (N = 302)^3^^,^^4^
**Inflammatory cytokines**		**pg/ml**	**pg/ml**	
**IL–1α /IL–1F1**	100%	24.7 (1.5–7093.6)	83.4 (401.1)	0.35; 0.51; 0.35
**IL–1β /IL–1F2**	93%	17.0 (0.5–19069.8)	204.5 (1330.4)	0.58; 0.52; 0.55
**IL–6**	95%	14.2 (0.7–3049.1)	68.0 (234.2)	0.50; 0.48; 0.53
**IL–12**^**5**^	77%	11.5 (0.1–356.1)	16.2 (26.7)	0.46; 0.55; 0.42
**IL–18 (n = 301)**	92%	21960.0 (125.0–1055770.0)	49244.1 (100235.8)	0.54; 0.46; 0.58
**TNF-α**^**5**^	41%	1.0 (0.2–75.2)	3.9 (9.8)	0.36; 0.52; 0.35
**Anti-inflammatory cytokines**		**pg/ml**	**pg/ml**	
**IL–10 (n = 301)**	86%	55.3 (3.5–660.5)	78.1 (78.3)	0.34; 0.43; 0.39
**Adaptive cytokines**		**pg/ml**	**pg/ml**	
**IFN-y**^**5**^	36%	7.1 (0.1–140.0)	12.7 (19.3)	0.31; 0.42; 0.37
**IL–2**	93%	24.8 (2.1–342.0)	30.5 (25.8)	0.30; 0.55; 0.27
**IL–4**^**5**^	49%	2.8 (0.3–20.9)	3.6 (3.1)	0.25; 0.40; 0.31
**IL–15**^**5**^	44%	0.8 (0.1–7.8)	1.1 (1.2)	0.33; 0.57; 0.29
**IL–16**^**5**^	69%	67.5 (3.7–1931.3)	128.2 (238.4)	0.36; 0.50; 0.36
**IL–17**^**5**^ **(N = 301)**	82%	32.7 (1.0–1203.1)	87.9 (162.9)	0.45; 0.49; 0.46
**CC Chemokines**		**pg/ml**	**pg/ml**	
**MIP–1α**^**5**^**/CCL3**	60%	7.8 (0.6–49.3)	10.1 (7.8)	0.27; 0.41; 0.33
**MIP–1β/CCL4**	89%	23.1 (0.5–760.6)	46.1 (83.5)	0.30; 0.41; 0.37
**MCP–1/CCL2**	88%	14.8 (1.8–6034.7)	75.4 (368.0)	0.38; 0.31; 0.57
**MCP–2**^**5**^**/CCL8**	63%	8.4 (0.2–74.0)	9.8 (8.5)	0.33; 0.44; 0.38
**RANTES/ CCL5**	86%	4.0 (0.2–555.2)	17.5 (56.8)	0.52; 0.54; 0.48
**CXC Chemokines**		**pg/ml**	**pg/ml**	
**IP–10/ CXCL10**	99%	287.5 (7.4–4686.0)	574.8 (768.2)	0.42; 0.52; 0.41
**SDF–1β/CXCL12**^**5**^	41%	71.7 (17.4–2071.5)	163.0 (295.9)	0.30; 0.51; 0.30
**MIG/CXCL9**^**5**^	75%	5354.9 (174.2–115344.4)	9778.1 (14319.5)	0.58; 0.61; 0.46
**IL–8**	100%	491.1 (4.6–82557.8)	1317.0 (4957.9)	0.37; 0.46; 0.40
**Growth Factors**		**pg/ml**	**pg/ml**	
**GM–CSF**	95%	4.4 (0.2–84.9)	5.9 (6.2)	0.31; 0.49; 0.32
**G–CSF**	92%	30.4 (2.2–225.6)	261.1 (476.0)	0.50; 0.55; 0.45
**TGF–α**	89%	40.1 (0.4–322.9)	36.8 (28.2)	0.33; 0.57; 0.28
**TGF–β**	99%	104.3 (0.8–4507.0)	54.5 (47.5)	0.34; 0.37; 0.45
**Antimicrobial proteins**		**ng/ml**	**ng/ml**	
**IFN–β**^**5**^	64%	0.3 (0.0–8.6)	0.4 (0.8)	0.42; 0.42; 0.50
**SLPI**	100%	238.5 (5.0–10892.2)	441.8 (869.3)	0.28; 0.33; 0.40
**Elafin**	100%	107.0 (9.6–7548.3)	185.7 (459.0)	0.22; 0.41; 0.26
**s100a8 (n = 302)**	100%	**9.9 (1.1–159.7)**	**13.9 (15.8)**	0.21; 0.37; 0.27
**HNP 1–3**	100%	342.9 (4.0–28582.2)	1635.1 (3703.9)	0.45; 0.38; 0.57
**HBD2 (n = 302)**	95%	446.2 (0.3–17689.2)	1374.6 (2435.7)	0.49; 0.38; 0.62
**HBD3 (n = 301)**	97%	7.8 (0.1–2807.2)	132.8 (410.9)	0.57; 0.32; 0.83
**HBD4 (n = 302)**	86%	0.4 (0.0–3.2)	0.5 (0.5)	0.32; 0.49; 0.33
**Immunoglobulins**		**ng/ml**	**ng/ml**	
**pIgR (n = 301)**	99%	68.9 (0.3–2198.1)	144.2 (250.5)	0.41; 0.42; 0.49
**IgA (n = 230)**	96%	401.1 (30.3–14977.4)	1016.9 (2024.7)	0.37; 0.36; 0.50
**IgM**^**5**^ **(n = 230)**	75%	137.6 (6.6–17905.0)	562.0 (1594.1)	0.51; 0.44; 0.57
**IgG1 (n = 230)**	99%	9123.7 (355.3–110238.7)	14815.8 (17047.5)	0.23; 0.14; 0.57
**IgG2 (n = 230)**	90%	3963.0 (325.8–96848.8)	7478.8 (10841.0)	0.41; 0.48; 0.43
**IgG3 (n = 230)**	97%	312.3 (18.6–18261.1)	666.5 (1466.2)	0.33; 0.20; 0.67
**IgG4 (n = 230)**	90%	80.7 (0.9–2662.5)	250.7 (422.5)	0.71; 0.68; 0.48
**Controls**		**ng/ml**	**ng/ml**	
**APOA1 (n = 302)**	100%	0.04 (0.0–78.6)	1.3 (6.0)	0.65; 0.36; 0.86
**SCCA–1 (n = 301)**	97%	122.7 (0.5–5072.3)	254.2 (503.8)	0.36; 0.31; 0.55
**Albumin (n = 302)**	100%	1802.4 (27.7–284126.9)	5761.3 (20514.8)	0.38; 0.46; 0.42
**Involucrin (n = 302)**	100%	50.1 (0.7–33668.8)	431.9 (2351.2)	0.47; 0.46; 0.51
**Total protein**	100%	123290.0 (27517.2–786809.2)	148872.1 (104461.5)	0.19; 0.42; 0.22

1. Samples with analyte concentrations below the lower limit of quantification (LLOQ) of the assay were assigned a concentration of half the LLOQ.

2. σ = variance between women; ICC = intra-class correlation coefficient; σ_w_ = within-woman variance.

3. Restricted to 302 samples in which involucrin was measured.

4. For all of the analytes, there is strong evidence of clustering within women—i.e. strong evidence that ICC >0; the p-value is <0.001 calculated by ANOVA, using an F test of between-women variation/within-women variation.

5. Biomarkers with <85% observations below the limit of quantification

Inter- and intra-woman variation in analyte concentration differed from one analyte to another (Table 2). For all analytes, there was strong evidence of a correlation between repeated samples within the same woman over time (p<0.001). For most analytes, the ICC was close to 0.50 (median = 0.45, IQR = 0.38–0.51).

#### Menstrual cycle phase signature

**Fig 2:**
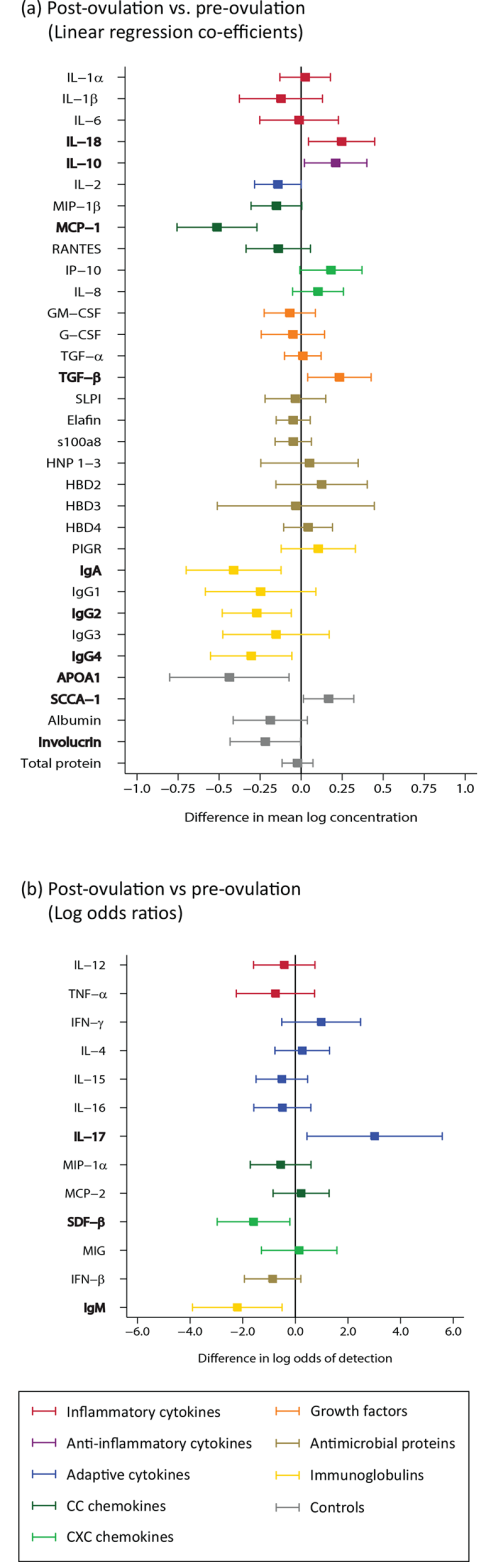
Menstrual cycle phase (n = 102). A comparison of analyte concentration between samples from visits occurring post-ovulation to pre-ovulation (reference). Menstrual cycle stage was assessed by measurement of urine pregnanediol 3-glucuronide. Bolded analytes represent associations with a p-value ≤0.05. (A) Analytes with ≥85% detection using linear regression showing coefficients (boxes) and confidence intervals (lines) (B) Analytes with <85% detection using logistic regression showing odds ratios (boxes) and confidence intervals (lines).

Compared to samples from visits during pre-ovulation, samples from visits during post-ovulation had increases in IL-18, IL-10, IL-17, and TGF-β, and a decrease in monocyte chemotactic protein (MCP)-1, stromal cell-derived factor (SDF)-1β, IgA, IgG2 and IgM (n = 102; Fig 2; Table B in S1 Table). There were no detectable difference in expression of antimicrobial proteins with menstrual cycle phase.

#### Hormonal contraception signatures

**Fig 3:**
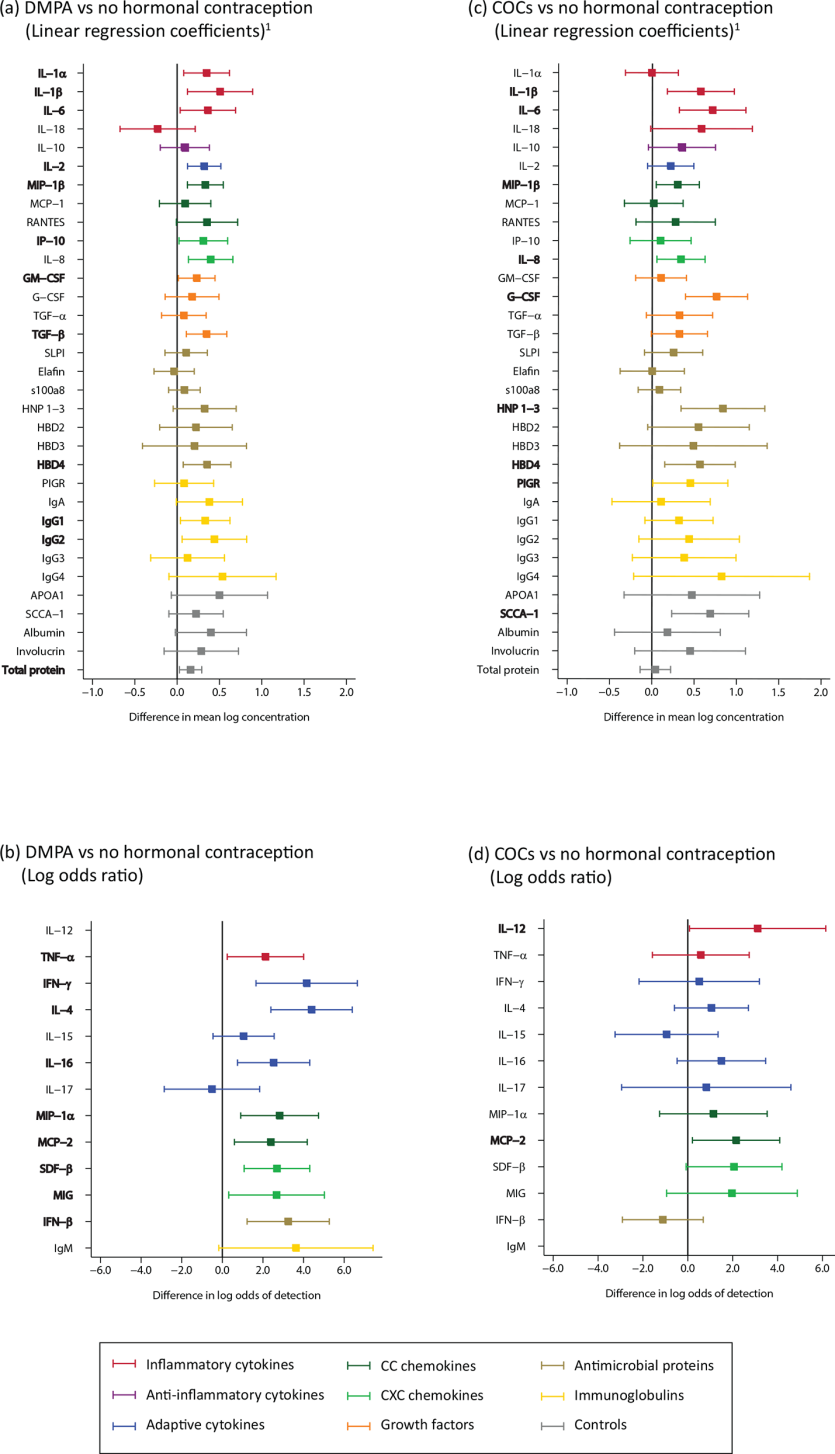
Reported hormonal contraceptive use (DMPA, n = 327; and COC, n = 305). Bolded analytes represent associations with a p-value ≤0.05. (A) A comparison of analyte concentration ≥85% between samples DMPA and women who reported no hormonal contraception use (reference) using linear regression. (B) A comparison of analyte concentration <85% between samples from women who reported use of DMPA and women who reported no hormonal contraception use (reference) using logistic regression. (C) A comparison of analyte concentration ≥85% between samples from women who reported use of COC and women who reported no hormonal contraception use (reference) using linear regression. (D) A comparison of analyte concentration <85% between samples from women who reported use of COC and women who reported no hormonal contraception use (reference) using logistic regression. Footnotes: 1. The x-axis range is from -1 to +2 which is wider than for all other figures; 2. For the association with DMPA and IL-12, odds ratios could not be estimated as all DMPA visits had detectable IL-12 levels; 3. For the association with COC use and IgM, odds ratios could not be estimated as all COC visits had detectable IgM levels.

Compared to women who reported no hormonal contraceptive use, women reporting DMPA use had an increase in concentration of several soluble proteins of the innate and adaptive immune system, including IL-1α, IL-1β, IL-6, TNF-α, IL-2, IL-4, IL-16, interferon (IFN)-γ, macrophage inflammatory protein (MIP)-1α, MIP-1β, MCP-2, IP-10, SDF-β, monokine induced by gamma interferon (MIG), IL-8, TGF-β, IFN-β, HBD4, IgA, IgG1, and IgG2 (n = 327; Fig 3A and 3B; Table B in S1 Table). Women reporting COC use had a similar signature with increases in IL-1β, IL-2, IL-6, IL-8, MIP-1β, MCP-2, G-CSF, HNP 1–3, HBD4, and SCCA-1 (n = 305; Fig 3C and 3D; Table B in S1 Table).

#### Signature for the detection of prostate-specific antigen

**Fig 4:**
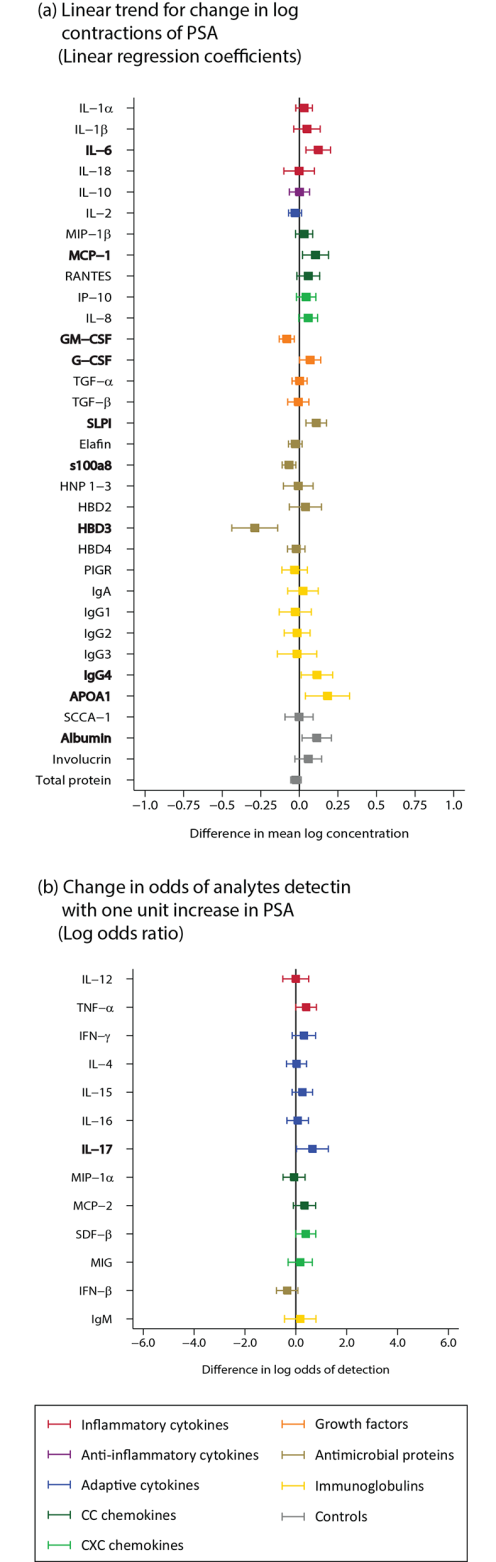
Prostate-specific antigen (PSA, n = 370). PSA categories: None, low positive (<4 ng/mL), high positive (≥4 ng/mL). Bolded analytes represent associations with a p-value ≤0.05. (A) Analytes with ≥85% detection using linear regression, linear trend for change in log concentration. (B) Analytes with <85% detection using logistic regression, change in odds of analyte detection (if <85% LLOQ) with one unit increase in exposure category.

There was modest evidence of a positive linear correlation between PSA levels and IL-6, MCP-1, SLPI, APOA1, and evidence of a negative linear correlation with s100a8, GM-CSF, and HBD3 (n = 370; Fig 4; Table B in S1 Table).

#### Intravaginal practice signatures

**Fig 5:**
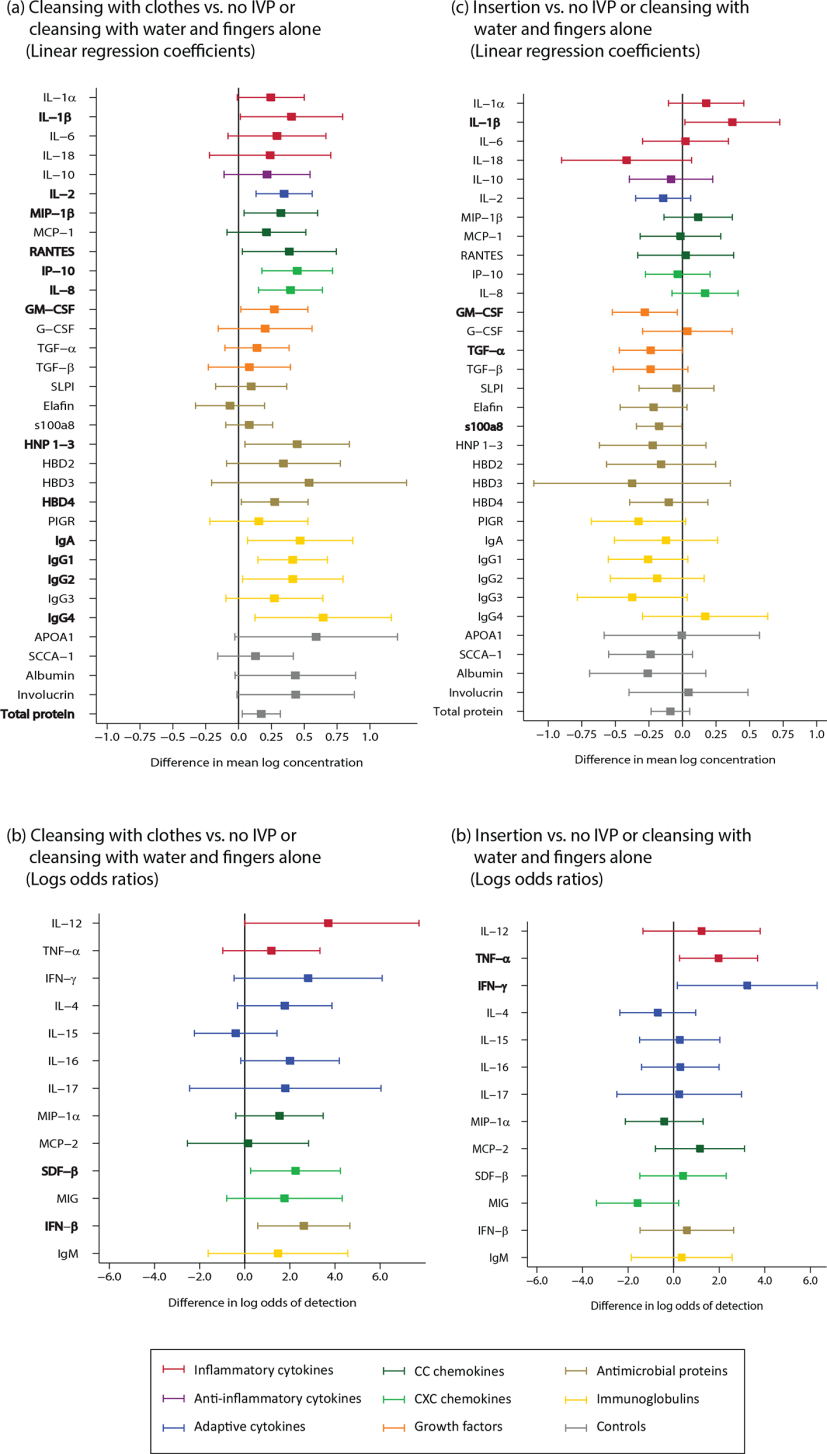
Traditional intravaginal practices (intravaginal cleansing with cloth, n = 155; and intravaginal insertion, n = 145). Bolded analytes represent associations with a p-value ≤0.05. (A) A comparison of analyte concentration ≥85% between samples from women who reported intravaginal cleansing with cloth and women who reported no intravaginal cleansing use or intravaginal cleansing with water alone (reference) using linear regression. (B) A comparison of analyte concentration <85% between samples from women who reported intravaginal cleansing with cloth and women who reported no intravaginal cleansing use or intravaginal cleansing with water alone (reference) using logistic regression. (C) A comparison of analyte concentration ≥85% between samples from women who reported intravaginal insertion and women who reported no intravaginal insertion (reference) using linear regression. (D) A comparison of analyte concentration <85% between samples from women who reported intravaginal insertion and women who reported no intravaginal insertion (reference) using logistic regression.

For the analysis of cleansing with soap, the analyte signature showed no evidence of a difference between women who used soap versus no cleansing or cleansing with water alone (Table B in S1 Table). For the analysis of cleansing with cloth, there was evidence of an increase in the innate and adaptive immune system proteins, including IL-1β, IL-2, IL-8, MIP-1β, SDF-1β, RANTES, IP-10, HNP 1–3, HBD4, IFN-β, and immunoglobulins (n = 155; Fig 5A and 5B; Table B in S1 Table). For the analysis of insertion, with exception of IL- β, TNF-α and IFN-γ, most of the other analytes trend towards a decrease for visits with reported insertion (n = 145; Fig 5C and 5D; Table B in S1 Table).

#### Analyte signatures from clinical findings

**Fig 6:**
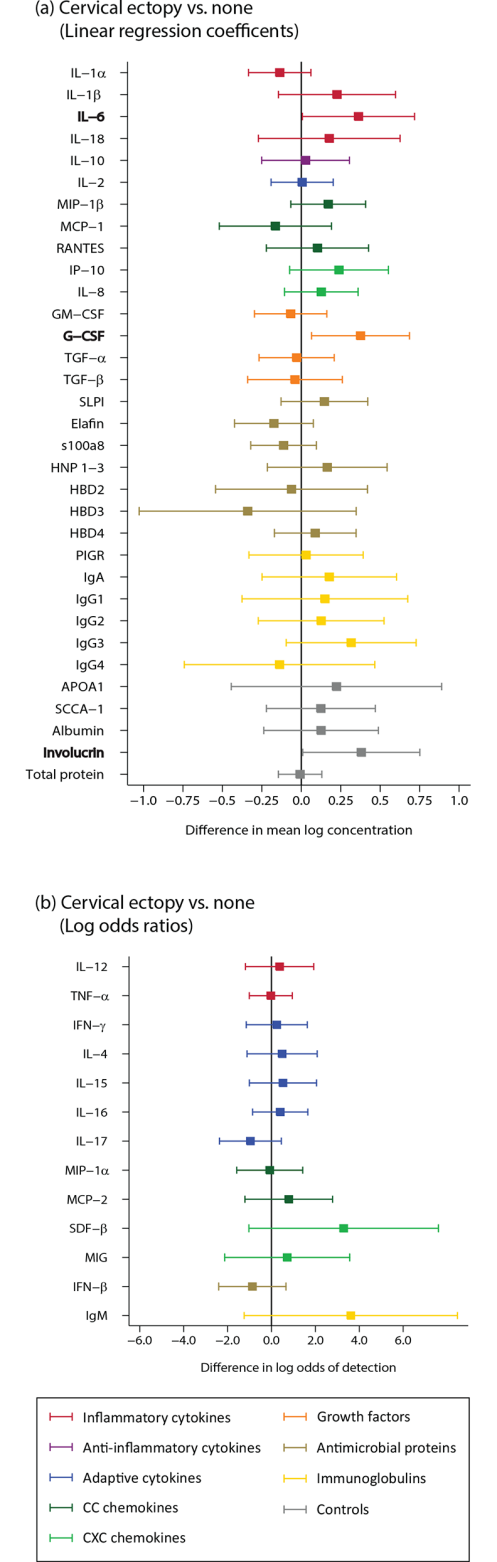
Clinical cervical ectopy (n = 67). Bolded analytes represent associations with a p-value ≤0.05. A comparison of analyte concentration between samples among women with cervical ectopy and women without ectopy (reference). (A) Analytes with >85% detection using linear regression; (B) Analytes with <85% detection using logistic regression.

For the analysis of cervical ectopy, we compared samples from women who had any ectopy at the first and last visit to samples from women who had no clinical ectopy. Samples from visits with clinical ectopy showed some evidence of an increase in IL-6 and G-CSF (n = 67; Fig 6; Table B in S1 Table).

**Fig 7:**
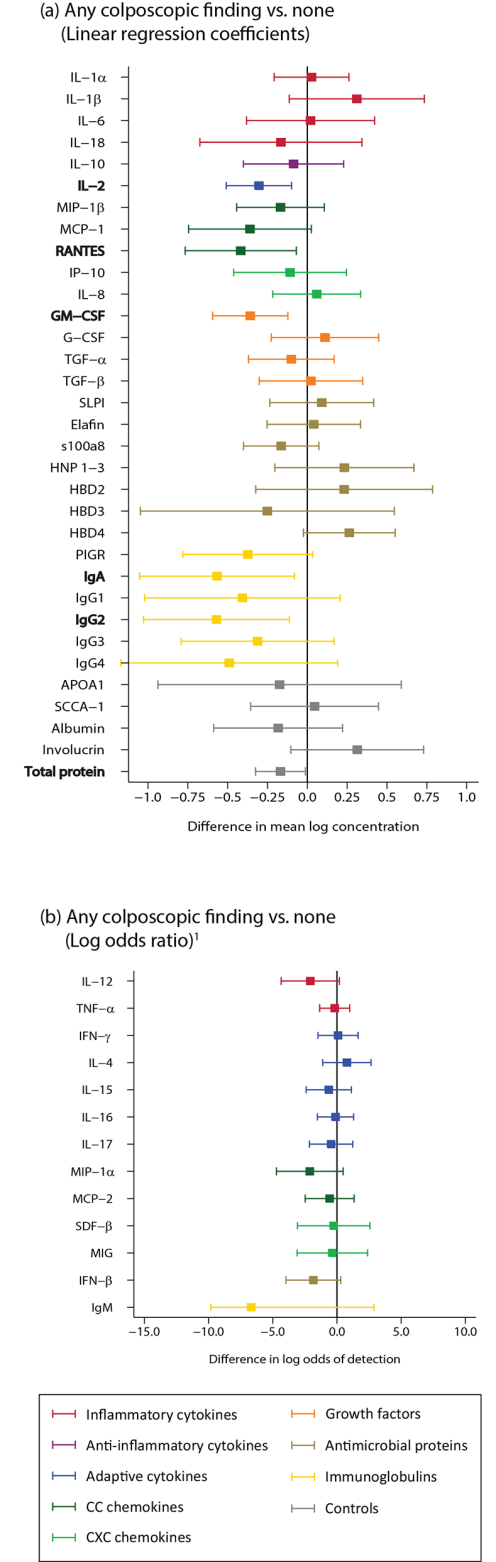
Colposcopy examination (n = 67). Bolded analytes represent associations with a p-value ≤0.05. A comparison of analyte concentration between samples among women with colposocopic findings and women without colposcopic findings (reference). (A) Analytes with >85% detection using linear regression; (B) Analytes with <85% detection using logistic regression. Footnote: 1. The x-axis range is from -15.0 to +10.0 which is wider than for all other figures.

For the analysis of colposcopy, we compared samples from women who had any colposcopic finding (88% [14/16] were petechiae) at the first and last visit to samples from women who had no colposcopic findings. Samples from visits with colposcopic findings showed a decrease in IL-2, RANTES, and GM-CSF compared to visits without colposcopic findings (n = 67; Fig 7; Table B in S1 Table).

**Fig 8:**
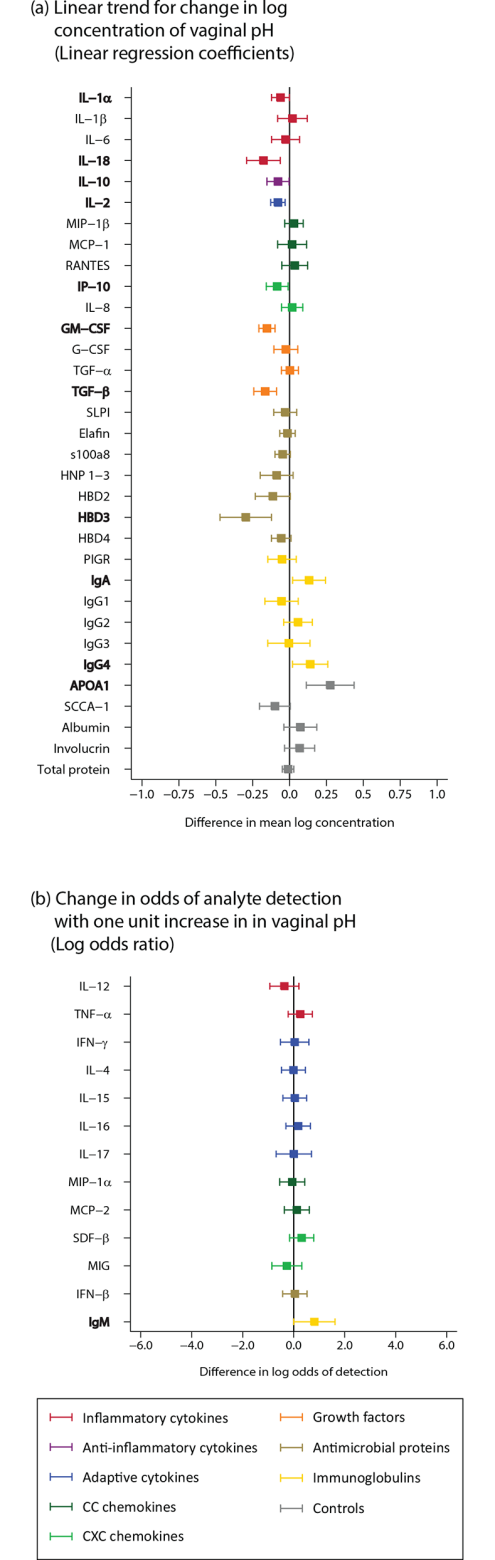
Vaginal pH (n = 361). Bolded analytes represent associations with a p-value ≤0.05. Vaginal pH was measured with test strips during the clinical examination: 3.6–4.1 (normal pH); 4.4–4.7 (high normal pH); and 5.0 and above (abnormal pH). (A) For analytes with >85% detection using linear regression, linear trend for change in log concentration. (B) Analytes with <85% detection using logistic regression, change in odds of analyte detection with one unit increase in exposure category.

There was some evidence for a negative linear correlation between pH and the concentration of inflammatory cytokine IL-18; adaptive cytokine IL-2; growth factors GM-CSF and TGF-β; and HBD3 (n = 361; Fig 8; Table B in S1 Table). There was evidence of a positive linear correlation of pH with IgA, IgG4 and APOA1.

#### Signatures from WBCs identified in the CVL cell pellet

**Fig 9:**
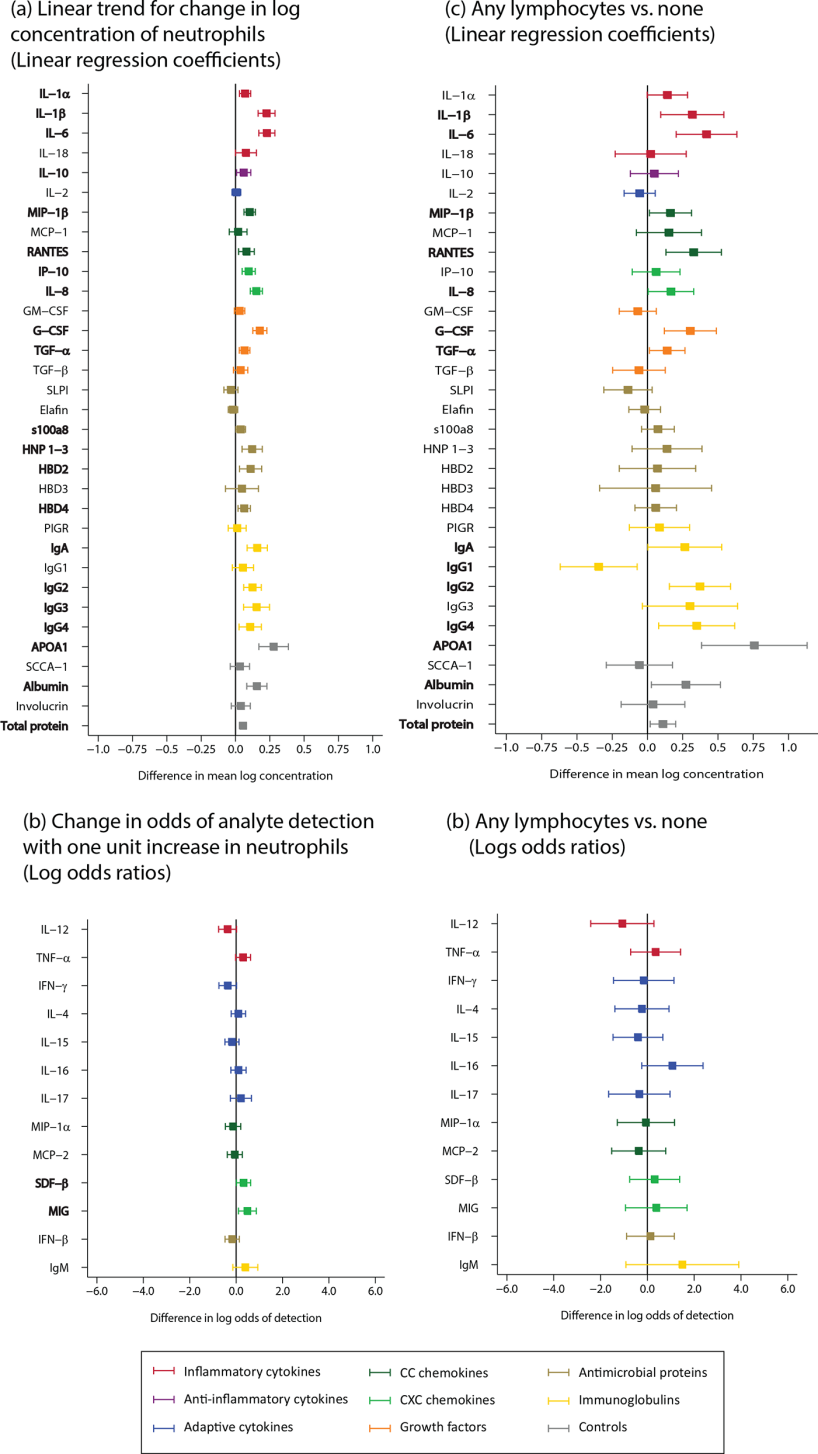
White blood cells (WBCs; neutrophils, n = 361; lymphocytes, n = 361). WBCs from the CVL cell pellet were identified and enumerated. For the statistical analysis, lymphocytes were either present or absent, and neutrophil were categorized as follows: no cells, 1–10 cells, 11–50 cells, >50 cells. Bolded analytes represent associations with a p-value ≤0.05. (A) For analytes with >85% detection using linear regression, linear trend for change in log concentration in neutrophils. (B) Analytes with <85% detection using logistic regression, change in odds of analyte detection with one unit increase in neutrophil category. (C) A comparison of analyte concentration >85% between samples with presence of lymphocytes and samples with absent lymphocytes (reference) using linear regression. (D) A comparison of analyte concentration >85% between samples with presence of lymphocytes and samples with absent lymphocytes (reference) using logistic regression.

We carried out analyses for neutrophils and lymphocytes only because there were too few other WBCs reported. There was evidence for strong positive linear correlation between neutrophil count and the concentration of analytes in the innate and adaptive immune system, including IL-1α, IL-1β, IL-6, MIP-1β, IP-10, IL-8, G-CSF, TGF-α, HNP 1–3, HBD2, HBD4, immunoglobulins, APOA1, albumin, and total protein (n = 361; Fig 9; Table B in S1 Table). For the analysis of lymphocytes, we compared samples that had any lymphocytes to those that had none. There was strong evidence of a positive association between samples that had detectable lymphocytes and IL-1β, IL-6, RANTES, G-CSF, IgG2, and APOA1, albumin and total protein, and a negative association between samples with detectable lymphocytes and IgG1 (n = 361; Fig 9; Table B in S1 Table).

#### Signatures from samples with haemoglobin

**Fig 10:**
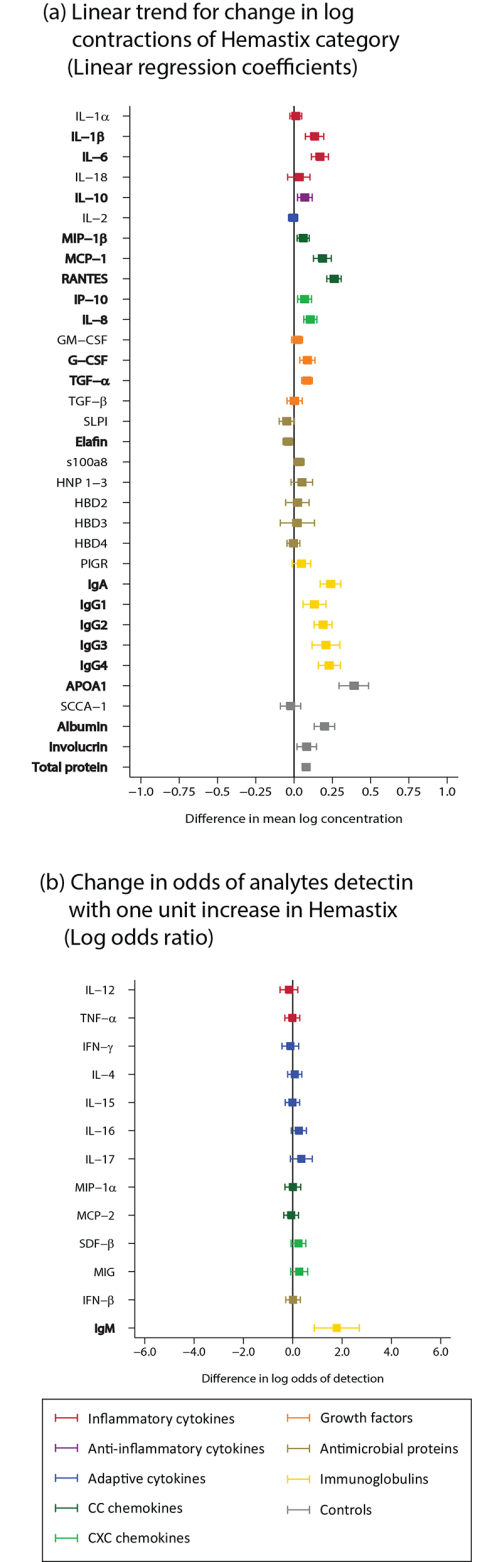
Haemoglobin (n = 369). Haemoglobin was measured by Hemostix test strips in the CVLs, categories were none, low (25 erythrocytes [ery]/μL), moderate (80 ery/μL), high (200 ery/μL). Bolded analytes represent associations with a p-value ≤0.05. (A) For analytes with >85% detection using linear regression, linear trend for change in log concentration. (B) Analytes with <85% detection using logistic regression, change in odds of analyte detection with one unit increase in exposure category.

There was evidence for strong positive linear correlation between haemoglobin category and concentration of IL-1β, IL-6, IL-10, MIP-1β, MCP-1, RANTES, IP-10, IL-8, G-CSF, TGF-α, immunoglobulins, APOA1, albumin and total protein (n = 369; Fig 10; Table B in S1 Table).

#### Adjustments for age, recent sex and samples with haemoglobin

We controlled for the effects of age, reported sexual intercourse in the past three days and presence of haemoglobin. All signatures showed little change after adjustment (Figs C-K in S1 Fig). Adjusted coefficients can been found in Table C in S1 Table.

## Discussion

We measured 45 different soluble immune proteins and peptides in CVLs from women taking part in a microbicide feasibility study, and who would be appropriate for enrolment into Phase 3 clinical trials for HIV prevention candidate products. During healthy visits, we found differential expression of soluble immune proteins by hormonal contraception use, menstrual cycle stage, recent sexual exposure, clinical findings, and some types of traditional intravaginal practices. Increasingly, inflammatory cytokines and other soluble immune proteins are being used to assess the potential safety of candidate microbicides, vaccines or other HIV prevention products [12,13]. These behavioural and biological factors should be taken into account in the design, analysis and interpretation of clinical trials using these analytes.

DMPA and COCs are the most popular forms of contraception in East and southern Africa [44]. A recent meta-analysis has suggested that DMPA, but not COCs, increase risk for HIV acquisition [45]. Our study showed an increase in innate and adaptive, inflammatory and anti-inflammatory analytes, signalling an overall heightened inflammatory state among women using DMPA and a similar signature for women using COCs. This is consistent with two recent studies showing increases in cytokines and chemokines among women using DMPA [46,47] and COCs [46]. Both studies suggest that the reported increase in RANTES among DMPA users may be a key factor underlying epidemiological associations between DMPA use and increased HIV acquisition; while COCs users showed no evidence of an increase in RANTES [46]. In our study, we see a trend towards an increase in RANTES among DMPA users; however, confidence intervals are wide. More studies are needed to investigate safety of effective contraception among women at high risk for HIV infection.

In contrast to hormonal contraception signatures, menstrual cycle phase signatures had relatively less differential expression. Consistent with our findings, both human and macaque studies have shown that IgA and IgM levels in the cervical mucus are decreased during the post-ovulation phase [48,49]. Published literature report differential expression of other soluble immune proteins by menstrual cycle phase [50], but the evidence is less consistent in *in vivo* studies. For example, concentrations of IL-1β and IL-6 have been found to be lower post-ovulation in one study of HIV-negative, pre-menopausal women in the US [24], but in a study among women living with HIV infection, levels of IL-1β and IL-6 were similar during pre-ovulation and post-ovulation phases [23]. In a study of healthy Belgian women, levels of IL-1β and IL-6 were also similar during pre-ovulation and post-ovulation phases, but there were increases in IL-1α and HBD1-3 post-ovulation. Additionally, in a small study that obtained vaginal biopsies from women pre-ovulation and post-ovulation, there were no differences in immune cell populations [51].

We measured PSA as a biomarker of recent seminal plasma exposure and found some evidence of differential expression with increasing PSA. A study of healthy Belgian women also reported a strong correlation between PSA and IL-6 [21]. Seminal plasma has been reported to stimulate pro-inflammatory cytokines and chemokines such as IL-8, IL-6, MCP-1 and GM-CSF in immortalized cervical cells, as well as stimulate infiltration of the cervix by macrophages, dendritic cells, and T lymphocytes [26]. It is thought that inflammation may facilitate preparation of the female reproductive tissues for pregnancy through clearance of pathogens, sperm selection, and induction of immune tolerance toward the semi-allogeneic embryo [52]. Additionally, epithelial microabrasions from sexual intercourse may cause immune activation; a small study reported that microabrasions were detected in 60% of healthy women after consensual intercourse [53].

Cervical ectopy typically occurs during times of increased oestrogen levels, including adolescence, pregnancy and among women taking oestrogen-containing hormonal contraceptives. Consistent with our results, Kyongo and colleagues also found a strong correlation between cervical ectopy and IL-6 and G-CSF, but also IL-1β, IL-8, MIP-1β among Belgian and sub-Saharan African women [21,22]. Additionally, Hwang and colleagues found that CVL samples among women with ectopy had high levels of IL-1β, IL-6 and IL-8, but also IL-1α, MIP-1α, RANTES, TNF-α, IL-10, IL-12 and IFN-γ [54]. There is evidence from published studies that ectopy is associated with COCs, but not with DMPA [55]. In our study, the signatures for COCs and ectopy are similar, and it may be that most of the differential expression seen among women with ectopy could be explained by increased levels of hormones (i.e. oestrogens).

Vaginal practices are very common in sub-Saharan Africa [56], and have been implicated in HIV acquisition and associated changes in vaginal microbiota [57]. In this population, intravaginal cleansing is highly prevalent, with many women using soap, while fewer women reported using cloth for cleansing or inserting substances [58]. Kyongo and colleagues report an increase in IL-6 and MIP-1 among women reporting using substances other than water for IVP in South Africa [22]. Our study showed no differential expression of analytes for intravaginal cleansing with soap; however, most analytes were *decreased* during visits with reported insertion use, and detergent was the most common substance inserted in our study. In previous *in vitro* studies, Nonoxynol-9 (N9), a surfactant (i.e. a detergent), has been shown to cause IL-1 release and activation of NF-kB in cervical and vaginal epithelial cells [8]. Our results suggest that soaps and detergents may not have a similar effect on the vaginal milieu as N9, though results should be interpreted with caution. In contrast, cloth use showed increased expression of the innate and adaptive, inflammatory and anti-inflammatory analytes, signalling an overall heightened inflammatory state among women using cloth. The mechanism behind this immune activation may be microabrasions caused by cloth use or introduction of bacteria. These results are consistent with epidemiological findings from a large individual person data analysis that showed an association between cloth use and HIV acquisition (pooled adjusted hazard ratio of 1.47, 95% confidence interval 1.18–1.83) indicating that cloth use may increase susceptibility to HIV acquisition [57]. More research is needed to understand the inflammatory effects of well-defined, prevalent IVP.

This study has a number of important strengths. We measured 45 different analytes, and reported concentrations found in CVLs in realistic trial conditions. We presented unadjusted raw data to show the differential expression of these analytes associated with biomedical and behavioural factors that are likely to be present during a clinical trial (e.g. hormonal contraception use, menstrual cycle stage, seminal plasma exposure). We have presented the data with figures showing unadjusted point estimates with confidence intervals in order to show signatures by factors. However, caution is needed when interpreting any one analyte estimate as this study has multiple comparisons using repeated significance testing which will lead to Type 1 errors or false positives. Therefore, it is more useful to look at the overall signatures, the patterns in types of analytes (e.g. inflammatory cytokines, immunoglobulins), and the strength of associations.

This study also has some limitations. Several biomedical and behavioural factors (e.g. menstrual phase, COCs, DMPA, rarer types of vaginal practices) had lower statistical power to detect associations and wide confidence intervals; therefore, caution is warranted in interpreting these results. However, even with lower power, signatures show marked immune activation for hormonal contraception and vaginal practices with cloth, and relatively less with menstrual cycle, intravaginal insertion, ectopy and colposcopic findings. Secondly, our methods for assigning menstrual phase did not identify the exact visit during ovulation, and samples were not collected during menstruation; therefore differential expression of analytes occurring during ovulation or menses only would have been missed. Lastly, we defined healthy visits by the absence of STIs measured in this study, and there may be unmeasured effects of other STIs (e.g. Human papillomavirus). Additionally, there may be unmeasured effects of persistent immune activation after clearance of HSV shedding [59].

## Conclusions

Soluble immune proteins may be useful biomarkers to assess immune activation to predict harm or indicate the risk for HIV-1 acquisition in clinical trials for vaginal microbicides, mucosal vaccines and other interventions. Biomedical and behavioural factors such as hormonal changes, by menstrual cycle stage or hormonal contraception use, recent sex, vaginal practices, and blood contamination may affect concentration of these proteins. Although, randomization should ensure that the timing of visits and presence or these factors are similar between trial arms, investigators should be aware of background variability and collect reliable data on these factors. Additionally, understanding the modulation of total antibody expression over a menstrual cycle should inform the assessment of specific antibody responses following a trial of a candidate HIV vaccine, and provide evidence for the standardization of obtaining samples during the menstrual cycle phase.

## Supporting Information

S1 Fig Figure A, Spearman rank correlations by analyte panel. Figure B, Conceptual model for exploring confounding. Figure C, Adjusted menstrual cycle phase signature. Figure D, Adjusted reported hormonal contraceptive signatures. Figure E, Adjusted prostate-specific antigen signature. Figure F, Adjusted traditional intravaginal practices signatures. Figure G, Adjusted clinical cervical ectopy signature. Figure H, Adjusted colposcopy findings signature. Figure I, Adjusted vaginal pH signature. Figure J, Adjusted white blood cells signature. Figure K, Adjusted haemoglobin signature. (DOCX)

S1 Table Table A, The intra–class correlation coefficients and standard deviations for raw data, protein normalized and involucrin normalized analyte data. Table B, Unadjusted coefficients for associations of analytes with biomedical and behavioural factors. Table C, Adjusted coefficients for associations of analytes with biomedical and behavioural factors. (DOCX)

S1 Text Normalisation to account for dilution factor in the cervicovaginal lavage. (DOCX)

## References

[1] UN Joint Programme on HIV/AIDS. Global Epidemic: UNAIDS Report on the Global AIDS Epidemic: 2012 [Internet]. Geneva; 2012. Available: http://www.unaids.org/en/media/unaids/contentassets/documents/epidemiology/2012/gr2012/20121120_UNAIDS_Global_Report_2012_en.pdf

[2] Abdool Karim Q, Abdool Karim SS, Frohlich JA, Grobler AC, Baxter C, Mansoor LE, et al Effectiveness and safety of tenofovir gel, an antiretroviral microbicide, for the prevention of HIV infection in women. Science (80-). 2010/07/21 ed. 2010;329: 1168–1174. [pii] 10.1126/science.1193748 PMC3001187 20643915

[3] Souza M De, Adams E, Benenson M, Gurunathan S, Tartaglia J, et al Vaccination with ALVAC and AIDSVAX to prevent HIV-1 infection in Thailand. N Engl J Med. 2009;361: 2209–20. 10.1056/NEJMoa0908492 19843557

[4] Grant RM, Lama JR, Anderson PL, McMahan V, Liu AY, Vargas L, et al Preexposure chemoprophylaxis for HIV prevention in men who have sex with men. N Engl J Med. 2010;363: 2587–99. 10.1056/NEJMoa1011205 21091279 PMC3079639

[5] Thigpen MC, Kebaabetswe PM, Paxton LA, Smith DK, Rose CE, Segolodi TM, et al Antiretroviral Preexposure Prophylaxis for Heterosexual HIV Transmission in Botswana. N Engl J Med. 2012; 120711140017009. 10.1056/NEJMoa1110711 22784038

[6] Baeten JM, Donnell D, Ndase P, Mugo NR, Campbell JD, Wangisi J, et al Antiretroviral Prophylaxis for HIV Prevention in Heterosexual Men and Women. N Engl J Med. 2012; 120711140017009. 10.1056/NEJMoa1108524 PMC3770474 22784037

[7] Choopanya K, Martin M, Suntharasamai P, Sangkum U, Mock PA, Leethochawalit M, et al Antiretroviral prophylaxis for HIV infection in injecting drug users in Bangkok, Thailand (the Bangkok Tenofovir Study): a randomised, double-blind, placebo-controlled phase 3 trial. Lancet. Elsevier Ltd; 2013;381: 2083–90. 10.1016/S0140-6736(13)61127-7 23769234

[8] Fichorova RN, Tucker LD, Anderson DJ. The molecular basis of nonoxynol-9-induced vaginal inflammation and its possible relevance to human immunodeficiency virus type 1 transmission. J Infect Dis. 2001/07/27 ed. 2001;184: 418–428. doi:JID010196 [pii] 10.1086/322047 11471099

[9] Mesquita PM, Cheshenko N, Wilson SS, Mhatre M, Guzman EM, Fakioglu E, et al Disruption of tight junctions by cellulose sulfate facilitates HIV infection: model of microbicide safety. J Infect Dis. 2009/07/10 ed. 2009;200: 599–608. 10.1086/600867 19586414 PMC2877491

[10] Duerr A, Huang Y, Buchbinder S, Coombs RW, Sanchez J, del Rio C, et al Extended follow-up confirms early vaccine-enhanced risk of HIV acquisition and demonstrates waning effect over time among participants in a randomized trial of recombinant adenovirus HIV vaccine (Step Study). J Infect Dis. 2012;206: 258–66. 10.1093/infdis/jis342 22561365 PMC3490694

[11] Haase AT. Targeting early infection to prevent HIV-1 mucosal transmission. Nature. Nature Publishing Group; 2010;464: 217–23. 10.1038/nature08757 20220840

[12] Cummins JE, Doncel GF. Biomarkers of cervicovaginal inflammation for the assessment of microbicide safety. Sex Transm Dis. 2009;36: S84–91. 10.1097/OLQ.0b013e3181994191 19218890

[13] Fichorova RN. Guiding the vaginal microbicide trials with biomarkers of inflammation. J Acquir Immune Defic Syndr. 2004;37 Suppl 3: S184–93. Available: http://www.pubmedcentral.nih.gov/articlerender.fcgi?artid=2643374&tool=pmcentrez&rendertype=abstract 16419271 PMC2643374

[14] Keller MJ, Guzman E, Hazrati E, Kasowitz A, Cheshenko N, Wallenstein S, et al PRO 2000 elicits a decline in genital tract immune mediators without compromising intrinsic antimicrobial activity. AIDS. 2007;21: 467–76. 10.1097/QAD.0b013e328013d9b5 17301565

[15] Bollen LJM, Blanchard K, Kilmarx PH, Chaikummao S, Connolly C, Wasinrapee P, et al No increase in cervicovaginal proinflammatory cytokines after Carraguard use in a placebo-controlled randomized clinical trial. J Acquir Immune Defic Syndr. 2008;47: 253–7. 10.1097/QAI.0b013e31815d2f12 18025996

[16] Moscicki A, Kaul R, Ma Y, Scott ME, Daud II, Bukusi EA, et al Measurement of mucosal biomarkers in a phase 1 trial of intravaginal 3% StarPharma LTD 7013 gel (VivaGel) to assess expanded safety. J Acquir Immune Defic Syndr. 2012;59: 134–40. 10.1097/QAI.0b013e31823f2aeb 22067666 PMC3261360

[17] Schwartz JL, Mauck C, Lai J-J, Creinin MD, Brache V, Ballagh SA, et al Fourteen-day safety and acceptability study of 6% cellulose sulfate gel: a randomized double-blind Phase I safety study. Contraception. 2006;74: 133–40. 10.1016/j.contraception.2006.02.008 16860051

[18] Buchbinder SP, Mehrotra DV, Duerr A, Fitzgerald DW, Mogg R, Li D, et al Efficacy assessment of a cell-mediated immunity HIV-1 vaccine (the Step Study): a double-blind, randomised, placebo-controlled, test-of-concept trial. Lancet. 2008;372: 1881–93. 10.1016/S0140-6736(08)61591-3 19012954 PMC2721012

[19] Gray GE, Allen M, Moodie Z, Churchyard G, Bekker L-G, Nchabeleng M, et al Safety and efficacy of the HVTN 503/Phambili study of a clade-B-based HIV-1 vaccine in South Africa: a double-blind, randomised, placebo-controlled test-of-concept phase 2b study. Lancet Infect Dis. 2011;11: 507–15. 10.1016/S1473-3099(11)70098-6 21570355 PMC3417349

[20] Fichorova RN, Lai J, Schwartz JL, Weiner DH, Mauck CK, Callahan MM. Baseline variation and associations between subject characteristics and five cytokine biomarkers of vaginal safety among healthy non-pregnant women in microbicide trials. Cytokine. Elsevier Ltd; 2011;55: 134–40. 10.1016/j.cyto.2011.03.016 21530305

[21] Kyongo JK, Jespers V, Goovaerts O, Michiels J, Menten J, Fichorova RN, et al Searching for lower female genital tract soluble and cellular biomarkers: defining levels and predictors in a cohort of healthy caucasian women. PLoS One. 2012;7: e43951 10.1371/journal.pone.0043951 22952818 PMC3432048

[22] Kyongo JK, Crucitti T, Menten J, Hardy L, Cools P, Michiels J, et al A cross-sectional analysis of selected genital tract immunological markers and molecular vaginal microbiota in Sub-Saharan African women with relevance to HIV risk and prevention. Clin Vaccine Immunol. 2015;22: CVI.00762–14. 10.1128/CVI.00762-14 PMC4412937 25761460

[23] Al-Harthi L, Kovacs a, Coombs RW, Reichelderfer PS, Wright DJ, Cohen MH, et al A menstrual cycle pattern for cytokine levels exists in HIV-positive women: implication for HIV vaginal and plasma shedding. AIDS. 2001;15: 1535–43. Available: http://www.ncbi.nlm.nih.gov/pubmed/11504986 11504986 10.1097/00002030-200108170-00011

[24] Al-Harthi L, Wright DJ, Anderson D, Cohen M, Matity Ahu D, Cohn J, et al The impact of the ovulatory cycle on cytokine production: evaluation of systemic, cervicovaginal, and salivary compartments. J Interferon Cytokine Res. 2000;20: 719–24. 10.1089/10799900050116426 10954915

[25] Huijbregts RPH, Helton ES, Michel KG, Sabbaj S, Richter HE, Goepfert PA, et al Hormonal contraception and HIV-1 infection: medroxyprogesterone acetate suppresses innate and adaptive immune mechanisms. Endocrinology. 2013;154: 1282–95. 10.1210/en.2012-1850 23354099 PMC3578997

[26] Sharkey DJ, Tremellen KP, Jasper MJ, Gemzell-Danielsson K, Robertson SA. Seminal fluid induces leukocyte recruitment and cytokine and chemokine mRNA expression in the human cervix after coitus. J Immunol. 2012;188: 2445–54. 10.4049/jimmunol.1102736 22271649

[27] Rebbapragada A, Howe K, Wachihi C, Pettengell C, Sunderji S, Huibner S, et al Bacterial vaginosis in HIV-infected women induces reversible alterations in the cervical immune environment. J Acquir Immune Defic Syndr. 2008/11/08 ed. 2008;49: 520–2. 10.1097/QAI.0b013e318189a7ca 18989228

[28] Fichorova RN, Desai PJ, Gibson FC, Genco CA. Distinct proinflammatory host responses to Neisseria gonorrhoeae infection in immortalized human cervical and vaginal epithelial cells. Infect Immun. 2001;69: 5840–8. 10.1128/IAI.69.9.5840 11500462 PMC98702

[29] Fichorova RN. Impact of T. vaginalis infection on innate immune responses and reproductive outcome. J Reprod Immunol. 2009;83: 185–9. 10.1016/j.jri.2009.08.007 19850356 PMC2788009

[30] Rebbapragada A, Wachihi C, Pettengell C, Sunderji S, Huibner S, Jaoko WG, et al Negative mucosal synergy between Herpes simplex type 2 and HIV in the female genital tract. AIDS. 2007;21: 589–98. 10.1097/QAD.0b013e328012b896 17314521

[31] Hilber AM, Chersich MF, van de Wijgert JHHM, Rees H, Temmerman M. Vaginal practices, microbicides and HIV: what do we need to know? Sex Transm Infect. 2007/11/21 ed. 2007;83: 505–508. 10.1136/sti.2007.028597 18024709 PMC2598638

[32] Kapiga SH, Ewings FM, Ao TT, Chilongani J, Mongi A, Baisley K, et al The Epidemiology of HIV and HSV-2 Infections among Women Participating in Microbicide and Vaccine Feasibility Studies in Northern Tanzania. PLoS One. 2013;8: e68825 10.1371/journal.pone.0068825 23874780 PMC3715536

[33] McCormack S, Ramjee G, Kamali A, Rees H, Crook AM, Gafos M, et al PRO2000 vaginal gel for prevention of HIV-1 infection (Microbicides Development Programme 301): a phase 3, randomised, double-blind, parallel-group trial. Lancet. 2010/09/21 ed. 2010;376: 1329–37. 10.1016/S0140-6736(10)61086-0 20851460 PMC2956883

[34] Watson-Jones D, Weiss HA, Rusizoka M, Changalucha JM, Baisley K, Mugeye KK, et al Effect of herpes simplex suppression on incidence of HIV among women in Tanzania. N Engl J Med. 2008/03/14 ed. 2008;358: 1560–71. 10.1056/NEJMoa0800260 18337596 PMC2643126

[35] Macaluso M, Lawson L, Akins R a, Valappil T, Hammond KR, Blackwell R, et al Prostate-specific antigen in vaginal fluid as a biologic marker of condom failure. Contraception. 1999;59: 195–201. Available: http://www.ncbi.nlm.nih.gov/pubmed/10382083 10382083 10.1016/s0010-7824(99)00013-x

[36] Christine Mauck. Manual for the Standardization of Colposcopy for the Evaluation of Vaginal Products [Internet]. 2004. Available: http://www.conrad.org/assets/attachments/Revised_Manual.PDF

[37] Doyle AM, Ross DA, Maganja K, Baisley K, Masesa C, Andreasen A, et al Long-term biological and behavioural impact of an adolescent sexual health intervention in Tanzania: follow-up survey of the community-based MEMA kwa Vijana Trial. PLoS Med. 2010;7: e1000287 10.1371/journal.pmed.1000287 20543994 PMC2882431

[38] Nugent RP, Krohn MA, Hillier SL, Vaginosis B. Reliability of diagnosing bacterial vaginosis is improved by a standardized method of gram stain interpretation. J Clin Microbiol. 1991;29: 297–301. Available: http://www.pubmedcentral.nih.gov/articlerender.fcgi?artid=269757&tool=pmcentrez&rendertype=abstract 1706728 10.1128/jcm.29.2.297-301.1991 PMC269757

[39] Hochmeister MN, Budowle B, Rudin O, Gehrig C, Borer U, Thali M, et al Evaluation of prostate-specific antigen (PSA) membrane test assays for the forensic identification of seminal fluid. J Forensic Sci. 1999;44: 1057–60. Available: http://www.ncbi.nlm.nih.gov/pubmed/10486959 10486959

[40] Biancotto A, Grivel J-C, Iglehart SJ, Vanpouille C, Lisco A, Sieg SF, et al Abnormal activation and cytokine spectra in lymph nodes of people chronically infected with HIV-1. Blood. 2007;109: 4272–9. 10.1182/blood-2006-11-055764 17289812 PMC1885500

[41] Fischetti L, Barry SM, Hope TJ, Shattock RJ. HIV-1 infection of human penile explant tissue and protection by candidate microbicides. AIDS. 2009;23: 319–28. 10.1097/QAD.0b013e328321b778 19114867 PMC4349942

[42] Kassam A, Overstreet JW, Snow-Harter C, De Souza MJ, Gold EB, Lasley BL. Identification of Anovulation and Transient Luteal Function Using a Urinary Pregnanediol-3-Glucuronide Ratio Algorithm. Environ Health Perspect. 1996;104: 408–413. 8732951 10.1289/ehp.96104408 PMC1469335

[43] O’Connor K a, Brindle E, Miller RC, Shofer JB, Ferrell RJ, Klein N a, et al Ovulation detection methods for urinary hormones: precision, daily and intermittent sampling and a combined hierarchical method. Hum Reprod. 2006;21: 1442–52. 10.1093/humrep/dei497 16439502

[44] United Nations. World Contraceptive Patterns 2013. United Nations, New York 2013; 11–12. 10.1016/S0140

[45] Morrison CS, Chen P-L, Kwok C, Baeten JM, Brown J, Crook AM, et al Hormonal contraception and the risk of HIV acquisition: an individual participant data meta-analysis. PLoS Med. 2015;12: e1001778 10.1371/journal.pmed.1001778 25612136 PMC4303292

[46] Morrison C, Fichorova R, Mauck C, Chen P, Kwok C, Chipato T, et al Cervical Inflammation and Immunity Associated with Hormonal Contraception, Pregnancy and HIV-1 Seroconversion. J Acquir Immune Defic Syndr. 2014; 10.1097/QAI.0000000000000103 24413042

[47] Deese J, Masson L, Miller W, Cohen M, Morrison C, Wang M, et al Injectable Progestin-Only Contraception is Associated With Increased Levels of Pro-Inflammatory Cytokines in the Female Genital Tract. Am J Reprod Immunol. 2015; n/a–n/a. 10.1111/aji.12415 26202107

[48] Usala SJ, Usala FO, Haciski R, Holt JA, Schumacher GF. IgG and IgA content of vaginal fluid during the menstrual cycle. J Reprod Med. 1989;34: 292–4. Available: http://www.ncbi.nlm.nih.gov/pubmed/2715991 2715991

[49] Lü FX, Ma Z, Rourke T, Srinivasan S, McChesney M, Miller CJ. Immunoglobulin concentrations and antigen-specific antibody levels in cervicovaginal lavages of rhesus macaques are influenced by the stage of the menstrual cycle. Infect Immun. 1999;67: 6321–8. Available: http://www.pubmedcentral.nih.gov/articlerender.fcgi?artid=97036&tool=pmcentrez&rendertype=abstract 10569744 10.1128/iai.67.12.6321-6328.1999 PMC97036

[50] Wira CR, Rodriguez-Garcia M, Shen Z, Patel M, Fahey JV. The role of sex hormones and the tissue environment in immune protection against HIV in the female reproductive tract. American Journal of Reproductive Immunology. 2014 pp. 171–181. 10.1111/aji.12235 24661500 PMC4106999

[51] Chandra N, Thurman AR, Anderson S, Cunningham TD, Yousefieh N, Mauck C, et al Depot medroxyprogesterone acetate increases immune cell numbers and activation markers in human vaginal mucosal tissues. AIDS Res Hum Retroviruses. 2013;29: 592–601. 10.1089/aid.2012.0271 23189932 PMC3581024

[52] Robertson SA. Seminal plasma and male factor signalling in the female reproductive tract. Cell Tissue Res. 2005;322: 43–52. 10.1007/s00441-005-1127-3 15909166

[53] Norvell MK, Benrubi GI, Thompson RJ. Investigation of microtrauma after sexual intercourse. J Reprod Med. 1984;29: 269–71. Available: http://www.ncbi.nlm.nih.gov/pubmed/6716372 6716372

[54] Hwang LY, Scott ME, Ma Y, Moscicki A-B. Higher levels of cervicovaginal inflammatory and regulatory cytokines and chemokines in healthy young women with immature cervical epithelium. J Reprod Immunol. 2011;88: 66–71. 10.1016/j.jri.2010.07.008 21051089 PMC3049722

[55] Bright PL, Norris Turner A, Morrison CS, Wong EL, Kwok C, Yacobson I, et al Hormonal contraception and area of cervical ectopy: a longitudinal assessment. Contraception. Elsevier Inc.; 2011;84: 512–9. 10.1016/j.contraception.2011.02.002 22018127 PMC4262923

[56] Hilber AM, Francis SC, Chersich MF, Scott P, Redmond S, Bender N, et al Intravaginal practices, vaginal infections and HIV acquisition: systematic review and meta-analysis. PLoS One. 2010/02/18 ed. 2010;5: e9119 10.1371/journal.pone.0009119 20161749 PMC2817741

[57] Low N, Chersich MF, Schmidlin K, Egger M, Francis SC, van de Wijgert JHHM, et al Intravaginal practices, bacterial vaginosis, and HIV infection in women: individual participant data meta-analysis. PLoS Med. 2011 Feb 1. 2011;8: e1000416 10.1371/journal.pmed.1000416 21358808 PMC3039685

[58] Francis SC, Baisley K, Lees SS, Andrew B, Zalwango F, Seeley J, et al Vaginal Practices among Women at High Risk of HIV Infection in Uganda and Tanzania: Recorded Behaviour from a Daily Pictorial Diary. Thorne C, editor. PLoS One. 2013;8: e59085 10.1371/journal.pone.0059085 23555618 PMC3608607

[59] Zhu J, Hladik F, Woodward A, Klock A, Peng T, Johnston C, et al Persistence of HIV-1 receptor-positive cells after HSV-2 reactivation is a potential mechanism for increased HIV-1 acquisition. Nat Med. 2009;15: 886–92. 10.1038/nm.2006 19648930 PMC2723183

