# Supplementary material for: Immune Activation in the Female Genital Tract: Expression Profiles of Soluble Proteins in Women at High Risk for HIV Infection
Source: PLoS One. 2016 Jan 27;11(1):e0143109. doi: 10.1371/journal.pone.0143109 (PMC4729472; doi:10.1371/journal.pone.0143109)
Supplement: S1 Fig — (DOCX) [file pone.0143109.s001.docx]

**Fig A**. **Spearman rank correlations by soluble immune protein panel to investigate potential for cross-reactivity by the beads during multiplex testing.** Shading indicates the correlation coefficients as indicated in the key.

**Fig B. Conceptual model for exploring confounding**. Changes in concentrations of analytes are likely to be affected by three broad categories: inflammation; hormonal changes; and blood contamination. Inflammation: We will not be able to account for all the causes for potential inflammation, but we hypothesized that recent sex, intravaginal practices, and ectopy could cause inflammation. Colposcopic findings (mostly petechaie) were probably due to recent sexual intercourse or intravaginal practices. Hormonal changes would be caused from the use of hormonal contraception or menstrual cycle stage. Blood contamination: Many of these analytes are found in the blood; therefore, the presence of blood may increase concentrations of analytes. WBCs may be from blood contamination (e.g. from menses), but are also the direct result of signaling from inflammatory cytokines.

**(A)** Post-ovulation vs pre-ovulation
(Linear regression coefficients)

**(B)** Post-ovulation vs pre-ovulation
(Odds ratios)

**Fig C.** **Menstrual cycle phase (n=102) adjusted for participant age, reported sexual intercourse in the past 3 days, presence of hemoglobin in CVL.** A comparison of analyte concentration between samples from visits occurring post-ovulation to pre-ovulation (reference). Menstrual cycle stage was assessed by measurement of urine pregnanediol 3-glucuronide. (**A**) Analytes with ≥85% detection using linear regression showing coefficients (boxes) and confidence intervals (lines) (**B**) Analytes with <85% detection using logistic regression showing odds ratios (boxes) and confidence intervals (lines).

(**C**) COCs vs no hormonal contraception
(Linear regression coefficients)^1^

(**A**) DMPA vs no hormonal contraception
 (Linear regression coefficients)^1^

**(D)** COCs vs no hormonal contraception (Odds ratios)3

(**B**) DMPA vs no hormonal contraception (Odds ratios)^2^

**Fig D.** **Reported hormonal contraceptive use (DMPA, n=327; and COCs, n=305) adjusted for participant age, reported sexual intercourse in the past 3 days, presence of haemoglobin in CVL.** (A) A comparison of analyte concentration ≥85% between samples from women who reported use of DMPA and women who reported no hormonal contraception use (reference) using linear regression. (B) A comparison of analyte concentration <85% between samples from women who reported use of DMPA and women who reported no hormonal contraception use (reference) using logistic regression. (C) A comparison of analyte concentration ≥85% between samples from women who reported use of COCs and women who reported no hormonal contraception use (reference) using linear regression. (D) A comparison of analyte concentration <85% between samples from women who reported use of COCs and women who reported no hormonal contraception use (reference) using logistic regression. Footnotes: 1. The x-axis range is from -1 to +2 which is wider than for all other figures; 2. For the association with DMPA and IL-12, odds ratios could not be estimated as all DMPA visits had detectable IL-12 levels; 3. For the association with COC use and IgM, odds ratios could not be estimated as all COC visits had detectable IgM levels.

(**B**) Change in odds of analyte detection with one unit increase in PSA (Odds ratios)

(**A**) Linear trend for change in log concentration of PSA (Linear regression coefficients)

**Fig E.** **Prostate-specific antigen (PSA, n=370) adjusted for participant age, and presence of haemoglobin in CVL.** Seminal plasma was detected by measuring prostate-specific antigen (PSA). PSA categories: None, low positive (<4 ng/mL), high positive (≥4 ng/mL). (A) Analytes with ≥85% detection using linear regression, linear trend for change in log concentration. (B) Analytes with <85% detection using logistic regression, change in odds of analyte detection (if <85% LLOQ) with one unit increase in exposure category.

(**C**) Insertion vs no IVP or cleansing with water and fingers alone
(Linear regression coefficients)

**(A)** Cleansing with clothes vs no IVP or cleansing with water and fingers alone
(Linear regression coefficients)

(**D**) Insertion vs no IVP or cleansing with water and fingers alone
(Odds ratios)

(**B**) Cleansing with clothes vs no IVP or cleansing with water and fingers alone
(Odds ratios)

**Fig F.** **Traditional intravaginal practices (intravaginal cleansing with cloth, n=155; and intravaginal insertion, N=145) adjusted for participant age, reported sexual intercourse in the past 3 days, presence of haemoglobin in CVL.** (**A**) A comparison of analyte concentration ≥85% between samples from women who reported intravaginal cleansing with cloth and women who reported no intravaginal cleansing use or intravaginal cleansing with water alone (reference) using linear regression. (**B**) A comparison of analyte concentration <85% between samples from women who reported intravaginal cleansing with cloth and women who reported no intravaginal cleansing use or intravaginal cleansing with water alone (reference) using logistic regression. (**C**) A comparison of analyte concentration ≥85% between samples from women who reported intravaginal insertion and women who reported no intravaginal insertion (reference) using linear regression. (**D**) A comparison of analyte concentration <85% between samples from women who reported intravaginal insertion and women who reported no intravaginal insertion (reference) using logistic regression.

(**B**) Any ectopy finding vs none
(Odds ratios)

(**A**) Any ectopy finding vs none
(Linear regression coefficients)

**Fig G.** **Clinical cervical ectopy (n=67) adjusted for participant age, reported sexual intercourse in the past 3 days, presence of haemoglobin in CVL.** A comparison of analyte concentration between samples among women with cervical ectopy and women without ectopy (reference). (A) Analytes with >85% detection using linear regression. (B) Analytes with <85% detection using logistic regression.

(**B**) Any colposcopic finding vs none
(Odds ratios)^1^

(**A**) Any colposcopic finding vs none
(Linear regression coefficients)

**Fig H. Colposcopy examination (n=67) adjusted for participant age, reported sexual intercourse in the past 3 days, presence of haemoglobin in CVL.** A comparison of analyte concentration between samples among women with colposocopic findings and women without colposcopic findings (reference). (A) Analytes with >85% detection using linear regression; (B) Analytes with <85% detection using logistic regression. Footnote: 1. The x-axis range is from -15.0 to +10.0 which is wider than for all other figures.

(**A**) Linear trend for change in log concentration of vaginal pH (Linear regression coefficients)

(**B**) Change in odds of analyte detection with one unit increase in vaginal pH (Odds ratios)

**Fig I.** **Vaginal pH (n=361) adjusted for participant age, reported sexual intercourse in the past 3 days, presence of haemoglobin in CVL.** Vaginal pH was measured with test strips during the clinical examination: 3.6-4.1 (normal pH); 4.4-4.7 (high normal pH); and 5.0 and above (abnormal pH). (A) For analytes with >85% detection using linear regression, linear trend for change in log concentration. (B) Analytes with <85% detection using logistic regression, change in odds of analyte detection with one unit increase in exposure category.

(**C**) Any lymphocytes vs none (Linear regression coefficients)

(**A**) Linear trend for change in log concentration of neutrophils (Linear regression coefficients)

(**D**) Any lymphocytes vs none (Odds ratios)

(**B**) Change in odds of analyte detection with one unit increase in neutrophils (Odds ratios)

**Fig J. White blood cells (WBCs; neutrophils, n=361; lymphocytes, N=361) adjusted for participant age, and presence of haemoglobin in CVL.** WBCs from the CVL cell pellet were identified and enumerated. For the statistical analysis, lymphocytes were either present or absent, and neutrophil were categorized as follows: no cells, 1–10 cells, 11–50 cells, >50 cells. (A) For analytes with >85% detection using linear regression, linear trend for change in log concentration in neutrophils. (B) Analytes with <85% detection using logistic regression, change in odds of analyte detection with one unit increase in neutrophil category. (C) A comparison of analyte concentration >85% between samples with presence of lymphocytes and samples with absent lymphocytes (reference) using linear regression. (D) A comparison of analyte concentration >85% between samples with presence of lymphocytes and samples with absent lymphocytes (reference) using logistic regression.

(**B**) Change in odds of analyte detection with one unit increase in Hemastix category
(Odds ratios)

(**A**) Linear trend for change in log concentration of Hemastix category
(Linear regression coefficients)

**Fig K.** **Hemoglobin (n=369) adjusted for participant age. Hemoglobin was measured by Hemostix test strips in the CVLs, categories were none, low (25 ery/μL), moderate (80 ery/μL), high (200 ery/μL).** (A) For analytes with >85% detection using linear regression, linear trend for change in log concentration. (B) Analytes with <85% detection using logistic regression, change in odds of analyte detection with one unit increase in exposure category.
