# Supplementary material for: Immune Activation in the Female Genital Tract: Expression Profiles of Soluble Proteins in Women at High Risk for HIV Infection
Source: PLoS One. 2016 Jan 27;11(1):e0143109. doi: 10.1371/journal.pone.0143109 (PMC4729472; doi:10.1371/journal.pone.0143109)
Supplement: S1 Table — (DOCX) [file pone.0143109.s002.docx]

**Table A.** The intra–class correlation coefficients (ICC) and standard deviations for raw data, protein normalized and involucrin normalized from 370 CVL samples from 67 healthy women at increased risk for HIV in Northwest Tanzania.

| **Analyte** | **σ ; ICC ; σ_w_ of log–transformed data (N=302)^1,2^** | | |
| --- | --- | --- | --- |
|  | Raw data | Protein normalized | Involucrin normalized |
| **Inflammatory cytokines** | |  |  |
| **IL–1α /IL–1F1** | 0.47; 0.48; 0.35 | 0.44; 0.47; 0.34 | 0.79; 0.56; 0.60 |
| **IL–1β /IL–1F2** | 0.76; 0.45; 0.57 | 0.68; 0.44; 0.52 | 0.84; 0.43; 0.71 |
| **IL–6** | 0.74; 0.53; 0.53 | 0.65; 0.47; 0.49 | 0.82; 0.51; 0.61 |
| **IL–12^3^** | 0.63; 0.56; 0.42 | 0.65; 0.52; 0.46 | 0.86; 0.62; 0.61 |
| **IL–18 (n=301)** | 0.82; 0.39; 0.59 | 0.77; 0.43; 0.55 | 1.07; 0.59; 0.71 |
| **TNF-α^3^** | 0.48; 0.44; 0.35 | 0.49; 0.39; 0.37 | 0.73; 0.60; 0.52 |
| **Anti-inflammatory cytokines** | |  |  |
| **IL–10 (n=301)** | 0.53; 0.40; 0.39 | 0.46; 0.42; 0.33 | 0.81; 0.66; 0.52 |
| **Adaptive cytokines** |  |  |  |
| **IFN-y^3^** | 0.50; 0.45; 0.37 | 0.56; 0.43; 0.41 | 0.80; 0.59; 0.57 |
| **IL–2** | 0.44; 0.53; 0.29 | 0.45; 0.43; 0.34 | 0.80; 0.61; 0.57 |
| **IL–4^3^** | 0.39; 0.38; 0.31 | 0.45; 0.34; 0.36 | 0.79; 0.58; 0.57 |
| **IL–15^3^** | 0.45; 0.60; 0.29 | 0.50; 0.56; 0.34 | 0.77; 0.66; 0.54 |
| **IL–16^3^** | 0.50; 0.48; 0.37 | 0.51; 0.45; 0.38 | 0.75; 0.58; 0.57 |
| **IL–17^3^ (N=301)** | 0.69; 0.44; 0.46 | 0.62; 0.44; 0.42 | 0.97; 0.61; 0.62 |
| **CC Chemokines** |  |  |  |
| **MIP–1α^3^/CCL3** | 0.42; 0.39; 0.33 | 0.48; 0.32; 0.38 | 0.75; 0.60; 0.56 |
| **MIP–1β/CCL4** | 0.47; 0.43; 0.37 | 0.46; 0.29; 0.39 | 0.68; 0.46; 0.56 |
| **MCP–1/CCL2** | 0.69; 0.45; 0.57 | 0.63; 0.39; 0.54 | 0.92; 0.58; 0.68 |
| **MCP–2^3^/CCL8** | 0.47; 0.33; 0.38 | 0.54; 0.25; 0.45 | 0.80; 0.55; 0.62 |
| **RANTES/ CCL5** | 0.71; 0.61; 0.47 | 0.62; 0.49; 0.46 | 0.75; 0.46; 0.59 |
| **CXC Chemokines** |  |  |  |
| **IP–10/ CXCL10** | 0.60; 0.52; 0.41 | 0.54; 0.37; 0.41 | 0.86; 0.49; 0.64 |
| **SDF–1β/CXCL12^3^** | 0.42; 0.51; 0.31 | 0.43; 0.38; 0.34 | 0.67; 0.58; 0.51 |
| **MIG/CXCL9^3^** | 0.73; 0.58; 0.47 | 0.64; 0.45; 0.48 | 0.91; 0.45; 0.69 |
| **IL–8** | 0.52; 0.45; 0.41 | 0.47; 0.39; 0.38 | 0.74; 0.46; 0.60 |
| **Growth Factors** |  |  |  |
| **GM–CSF** | 0.45; 0.46; 0.34 | 0.47; 0.41; 0.36 | 0.83; 0.56; 0.59 |
| **G–CSF** | 0.43; 0.57; 0.28 | 0.34; 0.56; 0.22 | 0.74; 0.63; 0.50 |
| **TGF–α** | 0.60; 0.32; 0.48 | 0.66; 0.36; 0.53 | 0.92; 0.52; 0.70 |
| **TGF–β** | 0.66; 0.54; 0.45 | 0.60; 0.50; 0.43 | 0.82; 0.56; 0.59 |
| **Antimicrobial proteins** | |  |  |
| **IFN–β^3^** | 0.67; **0.43**; 0.50 | 0.69; 0.36; 0.53 | 0.92; 0.59; 0.66 |
| **SLPI** | 0.49; 0.32; 0.40 | 0.50; 0.38; 0.41 | 0.79; 0.64; 0.54 |
| **Elafin** | 0.40; 0.57; 0.26 | 0.42; 0.70; 0.24 | 0.79; 0.68; 0.51 |
| **s100a8 (n=302)** | 0.34; 0.39; 0.27 | 0.32; 0.36; 0.25 | 0.71; 0.59; 0.51 |
| **HNP 1–3** | 0.74; 0.37; 0.57 | 0.63; 0.35; 0.49 | 0.80; 0.40; 0.62 |
| **HBD2 (n=302)** | 0.80; 0.34; 0.62 | 0.73; 0.34; 0.57 | 0.90; 0.49; 0.66 |
| **HBD3 (n=301)** | 1.03; 0.28; 0.84 | 0.96; 0.29; 0.79 | 1.25; 0.39; 0.97 |
| **HBD4 (n=302)** | 0.48; 0.62; 0.33 | 0.42; 0.57; 0.31 | 0.69; 0.46; 0.54 |
| **Immunoglobulins** |  |  |  |
| **pIgR (n=301)** | 0.65; 0.45; 0.49 | 0.58; 0.51; 0.44 | 0.89; 0.61; 0.61 |
| **IgA (n=230)** | 0.60; 0.41; 0.49 | 0.49; 0.26; 0.44 | 0.64; 0.40; 0.54 |
| **IgM (n=230)** | 0.76; 0.47; 0.57 | 0.62; 0.41; 0.49 | 0.77; 0.50; 0.59 |
| **IgG1** **(n=230)** | 0.61; 0.14; 0.57 | 0.56; 0.00; 0.55 | 0.81; 0.31; 0.71 |
| **IgG2 (n=230)** | 0.59; 0.48; 0.43 | 0.44; 0.43; 0.34 | 0.72; 0.53; 0.54 |
| **IgG3 (n=230)** | 0.74; 0.38; 0.65 | 0.72; 0.35; 0.66 | 0.96; 0.38; 0.83 |
| **IgG4 (n=230)** | 0.86; 0.69; 0.48 | 0.75; 0.65; 0.43 | 0.88; 0.63; 0.57 |
| **Controls** |  |  |  |
| **APOA1 (n=302)** | 1.07; 0.41; 0.85 | 0.96; 0.36; 0.78 | 0.98; 0.48; 0.78 |
| **SCCA–1 (n=301)** | 0.67; 0.28; 0.55 | 0.62; 0.25; 0.53 | 0.94; 0.50; 0.72 |
| **Total protein** | 0.28; 0.44; 0.22 |  | 0.63; 0.58; 0.48 |
| **Albumin (n=302)** | 0.55; 0.49; 0.42 | 0.42; 0.39; 0.35 | 0.61; 0.58; 0.47 |
| **Involucrin (n=302)** | 0.68; 0.61; 0.50 | 0.63; 0.58; 0.48 |  |

1. σ = variance between women; ICC= intra-class correlation coefficient ; σw= within-woman variance

2. Restricted to 302 samples in which involucrin was measured.

3. Biomarkers with <85% observations below the limit of quantification.

**Table B.** Unadjusted coefficients for associations of selected factors with concentration of 45 analytes and total protein from 370 CVL samples from 67 healthy women at increased risk for HIV in Northwest Tanzania ^1^

|  | | **Menstrual Cycle** | **Hormonal Contraception** Ref: No HC | | **Semen Exposure** Ref: Absent | **Intravaginal Practices** Ref: Cleansing with water and fingers only | | | **Clinical Findings** Ref: Absent | | |
| --- | --- | --- | --- | --- | --- | --- | --- | --- | --- | --- | --- |
| **Analytes** | | PDG | DMPA | OCPs | PSA^2, 3^ | Soap use | Cloth use | Insertion | Ectopy | Colposcopic findings | pH^2,4^ |
| Inflammatory cytokines | IL–1α /IL–1F1 | 0.02 | 0.35 | -0.01 | 0.03 | 0.11 | 0.25 | 0.18 | -0.14 | 0.03 | -0.06 |
|  | IL–1β /IL–1F2 | -0.12 | 0.51 | 0.57 | 0.05 | 0.10 | 0.40 | 0.37 | 0.23 | 0.31 | 0.02 |
|  | IL–6 | -0.01 | 0.36 | 0.71 | 0.12 | 0.09 | 0.29 | 0.02 | 0.36 | 0.02 | -0.03 |
|  | IL–12^7^ | 0.66 | -- | -- | 1.00 | 1.24 | 40.52 | 3.41 | 1.45 | 0.13 | 0.69 |
|  | IL–18 | 0.25 | -0.23 | 0.58 | 0.00 | -0.02 | 0.24 | -0.42 | 0.18 | -0.17 | -0.18 |
|  | TNF-α^7^ | 0.47 | 8.35 | 1.78 | 1.50 | 1.18 | 3.26 | 7.18 | 0.97 | 0.84 | 1.29 |
| Anti-inflammatory | IL–10 | 0.21 | 0.09 | 0.35 | 0.00 | 0.07 | 0.22 | -0.08 | 0.03 | -0.09 | -0.08 |
| Adaptive cytokines | IFNy^7^ | 2.67 | 63.62 | 1.66 | 1.37 | 2.55 | 16.58 | 25.25 | 1.27 | 1.09 | 1.04 |
|  | IL–2 | -0.14 | 0.32 | 0.22 | -0.03 | 0.02 | 0.35 | -0.14 | 0.01 | -0.30 | -0.08 |
|  | IL–4^7^ | 1.30 | 81.08 | 2.87 | 1.03 | 0.60 | 5.88 | 0.50 | 1.63 | 2.17 | 0.99 |
|  | IL–15^7^ | 0.60 | 2.85 | 0.39 | 1.29 | 0.63 | 0.67 | 1.31 | 1.69 | 0.53 | 1.04 |
|  | IL–16^7^ | 0.61 | 12.49 | 4.47 | 1.07 | 1.17 | 7.44 | 1.34 | 1.49 | 0.89 | 1.19 |
|  | IL–17^7^ | 20.31 | 0.60 | 2.28 | 1.92 | 1.41 | 6.03 | 1.28 | 0.38 | 0.63 | 1.00 |
| CC chemokines | MIP–1α/CCL3^7^ | 0.57 | 16.79 | 3.13 | 0.93 | 0.63 | 4.66 | 0.66 | 0.93 | 0.12 | 0.94 |
|  | MIP–1β/CCL4 | -0.15 | 0.33 | 0.30 | 0.03 | 0.04 | 0.32 | 0.12 | 0.17 | -0.17 | 0.03 |
|  | MCP–1/CCL2 | -0.51 | 0.09 | 0.02 | 0.10 | 0.14 | 0.21 | -0.02 | -0.16 | -0.36 | 0.02 |
|  | MCP–2/CCL8^7^ | 1.24 | 10.89 | 8.60 | 1.40 | 0.70 | 1.15 | 3.18 | 2.20 | 0.56 | 1.13 |
|  | RANTES/ CCL5 | -0.14 | 0.35 | 0.28 | 0.06 | 0.14 | 0.39 | 0.02 | 0.10 | -0.42 | 0.04 |
| CXC chemokines | IP–10/ CXCL10 | 0.18 | 0.31 | 0.10 | 0.04 | 0.16 | 0.45 | -0.04 | 0.24 | -0.11 | -0.08 |
|  | SDF–1β/CXCL12^7^ | 0.20 | 14.73 | 7.84 | 1.47 | 1.48 | 9.50 | 1.51 | 26.87 | 0.77 | 1.37 |
|  | MIG/CXCL9^7^ | 1.16 | 14.45 | 7.15 | 1.19 | 0.82 | 5.82 | 0.21 | 2.05 | 0.70 | 0.76 |
|  | IL–8 | 0.10 | 0.40 | 0.34 | 0.06 | 0.11 | 0.40 | 0.17 | 0.13 | 0.06 | 0.02 |
| Growth factors | GM–CSF | -0.07 | 0.23 | 0.10 | -0.08 | -0.02 | 0.27 | -0.28 | -0.07 | -0.36 | -0.15 |
|  | G–CSF | -0.05 | 0.18 | 0.76 | 0.07 | 0.09 | 0.20 | 0.04 | 0.38 | 0.11 | -0.03 |
|  | TGF-α | 0.01 | 0.08 | 0.32 | 0.00 | -0.01 | 0.14 | -0.24 | -0.03 | -0.10 | 0.00 |
|  | TGF-β | 0.23 | 0.35 | 0.32 | -0.01 | 0.03 | 0.08 | -0.24 | -0.04 | 0.02 | -0.17 |
| Antimicrobial proteins | IFN-β^7^ | 0.42 | 25.60 | 0.33 | 0.71 | 1.28 | 13.70 | 1.79 | 0.42 | 0.16 | 1.05 |
|  | SLPI | -0.03 | 0.11 | 0.25 | 0.11 | -0.02 | 0.10 | -0.04 | 0.15 | 0.09 | -0.03 |
|  | Elafin | -0.05 | -0.04 | 0.00 | -0.03 | -0.03 | -0.06 | -0.22 | -0.17 | 0.04 | -0.01 |
|  | s100a8 | -0.05 | 0.09 | 0.09 | -0.07 | 0.04 | 0.08 | -0.17 | -0.11 | -0.16 | -0.05 |
|  | HNP 1–3 | 0.05 | 0.32 | 0.84 | -0.01 | 0.05 | 0.45 | -0.22 | 0.16 | 0.23 | -0.09 |
|  | HBD2 | 0.12 | 0.22 | 0.55 | 0.04 | 0.12 | 0.34 | -0.16 | -0.06 | 0.23 | -0.11 |
|  | HBD3 | -0.03 | 0.21 | 0.49 | -0.29 | -0.08 | 0.54 | -0.38 | -0.34 | -0.25 | -0.30 |
|  | HBD4 | 0.04 | 0.35 | 0.57 | -0.02 | 0.07 | 0.28 | -0.10 | 0.09 | 0.26 | -0.06 |
| Immunoglobulins | PIGR | 0.10 | 0.08 | 0.45 | -0.03 | 0.00 | 0.15 | -0.33 | 0.03 | -0.11 | -0.05 |
|  | IgA | -0.41 | 0.38 | 0.11 | 0.02 | 0.16 | 0.47 | -0.12 | 0.18 | -0.31 | 0.13 |
|  | IgM^7^ | 0.11 | -- | -- | 1.19 | 6.09 | 4.36 | 1.43 | 37.19 | 0.03 | 2.25 |
|  | IgG1 | -0.25 | 0.33 | 0.32 | -0.03 | 0.10 | 0.41 | -0.26 | 0.15 | -0.15 | -0.05 |
|  | IgG2 | -0.27 | 0.44 | 0.44 | -0.01 | 0.09 | 0.41 | -0.19 | 0.13 | -0.31 | 0.06 |
|  | IgG3 | -0.15 | 0.12 | 0.38 | -0.01 | 0.02 | 0.27 | -0.37 | 0.32 | -0.05 | 0.00 |
|  | IgG4 | -0.30 | 0.54 | 0.82 | 0.11 | 0.17 | 0.64 | 0.17 | -0.14 | -0.23 | 0.14 |
| Controls | APOA1 | -0.44 | 0.50 | 0.47 | 0.18 | 0.17 | 0.59 | -0.01 | 0.22 | -0.17 | 0.28 |
|  | SCCA–1 | 0.17 | 0.22 | 0.69 | 0.00 | -0.10 | 0.13 | -0.24 | 0.12 | 0.05 | -0.10 |
|  | Albumin | -0.19 | 0.40 | 0.18 | 0.11 | 0.00 | 0.43 | -0.26 | 0.13 | -0.18 | 0.07 |
|  | Involucrin | -0.22 | 0.29 | 0.45 | 0.06 | 0.12 | 0.44 | 0.04 | 0.38 | 0.31 | 0.07 |
|  | Total protein | -0.02 | 0.16 | 0.04 | -0.02 | 0.07 | 0.17 | -0.09 | -0.01 | -0.17 | -0.01 |

**1**. Associations with analytes with ≥85% LLOQ modelled using random effects linear regression; coefficients represent change from reference group in log concentration. Associations with analytes with <85% LLOQ modelled using random effects logistic regression; coefficients represent odds ratios for detectable analytes concentration compared with reference group. **2**. Linear trend for change in log concentration (if ≥85% LLOQ)/change in odds of analyte detection (if <85% LLOQ) with one unit increase in exposure category. **3**. PSA categories: None, low positive (<4 ng/mL), high positive (≥4 ng/mL). **4.** Vaginal pH categories: 3.6-4.1; 4.4-4.7; and 5.0 and above. **5**. Neutrophil categories: No cells, 1–10 cells, 11–50 cells, >50 cells. **6**. Haemoglobin categories: None, low (25 ery/μL), moderate (80 ery/μL), high (200 ery/μL). **7.** Analytes with <85% observation LLOQ.

| Colour legend: | p≥0.1 |  | p<0.10 |  | p<0.05 |  | P<0.01 |  | P<0.005 |  | P<0.001 |  |
| --- | --- | --- | --- | --- | --- | --- | --- | --- | --- | --- | --- | --- |

**Table C.** Coefficients for associations of selected factors with concentration of 45 analytes and total protein adjusted **for *age, presence of haemoglobin and reported sex in past 3 days*** from 370 CVL samples from 67 healthy women at increased risk for HIV in Northwest Tanzania ^1^

|  | | **Menstrual Cycle** | **Hormonal Contraception** Ref: No HC | | **Semen Exposure** Ref: Absent | **Intravaginal Practices** Ref: Cleansing with water and fingers only | | | **Clinical findings** Ref: Absent | | |
| --- | --- | --- | --- | --- | --- | --- | --- | --- | --- | --- | --- |
| **Analytes** | | PDG | DMPA | OCPs | PSA^2, 3^ | Soap use | Cloth use | Insertion | Ectopy | Colposcopic findings | pH^2,4^ |
| **Inflammatory cytokines** | IL–1α /IL–1F1 | 0.05 | 0.31 | -0.03 | 0.03 | 0.08 | 0.17 | 0.12 | -0.13 | -0.05 | -0.06 |
|  | IL–1β /IL–1F2 | -0.05 | 0.48 | 0.56 | 0.06 | 0.05 | 0.32 | 0.37 | 0.20 | 0.36 | 0.02 |
|  | IL–6 | 0.07 | 0.27 | 0.70 | 0.13 | 0.04 | 0.20 | 0.03 | 0.39 | 0.12 | -0.02 |
|  | IL–12^7^ | 0.50 | -- | 22.54 | 9.85E-01 | 1.38 | 36.41 | 2.79 | 1.20 | 0.08 | 0.64 |
|  | IL–18 | 0.27 | -0.25 | 0.42 | 0.01 | -0.04 | 0.30 | -0.45 | 0.00 | -0.27 | -0.18 |
|  | TNF-α^7^ | 0.61 | 8.68 | 1.93 | 1.46 | 1.15 | 3.12 | 8.25 | 0.92 | 0.71 | 1.35 |
| Anti-inflam-matory | IL–10 | 0.24 | 0.07 | 0.30 | 0.01 | 0.05 | 0.22 | -0.07 | -0.02 | 0.03 | -0.08 |
| Adaptive cytokines | IFNy^7^ | 2.78 | 73.01 | 1.68 | 1.37 | 2.60 | 18.48 | 32.11 | 1.17 | 1.11 | 1.06 |
|  | IL–2 | -0.15 | 0.35 | 0.23 | -0.03 | 0.03 | 0.35 | -0.16 | -0.04 | -0.34 | -0.08 |
|  | IL–4^7^ | 1.79 | 88.94 | 3.14 | 0.99 | 0.56 | 8.69 | 0.52 | 1.62 | 2.51 | 1.05 |
|  | IL–15^7^ | 0.57 | 3.55 | 0.56 | 1.28 | 0.63 | 0.59 | 1.69 | 1.44 | 0.16 | 1.00 |
|  | IL–16^7^ | 0.68 | 13.39 | 4.42 | 1.05 | 1.18 | 6.18 | 1.44 | 1.51 | 1.38 | 1.20 |
|  | IL–17^7^ | 25.25 | 0.37 | 1.64 | 2.02 | 0.89 | 4.94 | 0.84 | 0.39 | 0.37 | 1.03 |
| CC chemokines | MIP–1α/CCL3^7^ | 0.53 | 16.41 | 3.12 | 0.91 | 0.64 | 4.48 | 0.65 | 0.93 | 0.12 | 0.98 |
|  | MIP–1β/CCL4 | -0.14 | 0.36 | 0.28 | 0.03 | 0.06 | 0.36 | 0.18 | 0.18 | -0.09 | 0.03 |
|  | MCP–1/CCL2 | -0.44 | 0.04 | 0.03 | 0.11 | 0.07 | 0.11 | 0.01 | -0.14 | -0.33 | 0.00 |
|  | MCP–2/CCL8^7^ | 1.47 | 11.71 | 9.59 | 1.35 | 0.56 | 0.72 | 2.33 | 4.68 | 0.14 | 1.23 |
|  | RANTES/ CCL5 | -0.06 | 0.33 | 0.25 | 0.07 | 0.10 | 0.34 | 0.13 | 0.10 | -0.21 | 0.03 |
| CXC chemokines | IP–10/ CXCL10 | 0.19 | 0.27 | 0.12 | 0.05 | 0.11 | 0.37 | -0.03 | 0.19 | -0.22 | -0.09 |
|  | SDF–1β/CXCL12^7^ | 0.16 | 15.66 | 7.40 | 1.48 | 1.47 | 9.26 | 1.80 | 94.48 | 5.62 | 1.41 |
|  | MIG/CXCL9^7^ | 1.12 | 11.50 | 6.94 | 1.15 | 0.74 | 3.03 | 0.24 | 4.17 | 0.00 | 0.76 |
|  | IL–8 | 0.14 | 0.33 | 0.31 | 0.06 | 0.09 | 0.33 | 0.19 | 0.09 | 0.09 | 0.03 |
| Growth factors | GM–CSF | -0.06 | 0.24 | 0.12 | -0.08 | -0.01 | 0.26 | -0.27 | -0.13 | -0.32 | -0.16 |
|  | G–CSF | -0.03 | 0.09 | 0.73 | 0.07 | 0.07 | 0.16 | 0.02 | 0.39 | 0.20 | -0.02 |
|  | TGF-α | 0.04 | 0.03 | 0.32 | 0.00 | -0.05 | 0.09 | -0.22 | -0.15 | -0.02 | 0.00 |
|  | TGF-β | 0.23 | 0.34 | 0.32 | -0.01 | 0.02 | 0.02 | -0.29 | -0.13 | -0.05 | -0.16 |
| Antimicrobial proteins | IFN-β^7^ | 0.35 | 26.03 | 0.32 | 0.70 | 1.39 | 17.99 | 2.16 | 0.30 | 0.14 | 1.06 |
|  | SLPI | -0.04 | 0.05 | 0.18 | 0.11 | -0.03 | 0.17 | -0.10 | 0.07 | -0.16 | -0.02 |
|  | Elafin | -0.06 | 0.00 | -0.04 | -0.03 | -0.07 | -0.09 | -0.27 | -0.25 | -0.04 | -0.01 |
|  | s100a8 | -0.04 | 0.08 | 0.05 | -0.06 | 0.03 | 0.08 | -0.13 | -0.18 | -0.16 | -0.05 |
|  | HNP 1–3 | 0.10 | 0.29 | 0.71 | 0.00 | 0.00 | 0.42 | -0.30 | 0.13 | 0.18 | -0.09 |
|  | HBD2 | 0.14 | 0.20 | 0.36 | 0.05 | 0.07 | 0.33 | -0.25 | -0.11 | -0.01 | -0.12 |
|  | HBD3 | 0.04 | 0.10 | 0.33 | -0.28 | -0.09 | 0.47 | -0.37 | -0.61 | -0.27 | -0.29 |
|  | HBD4 | 0.06 | 0.36 | 0.51 | -0.02 | 0.03 | 0.24 | -0.09 | 0.02 | 0.27 | -0.06 |
| Immunoglobulins | PIGR | 0.14 | -0.03 | 0.48 | -0.03 | -0.02 | 0.14 | -0.43 | 0.00 | 0.09 | -0.05 |
|  | IgA | -0.32 | 0.24 | 0.21 | 0.02 | 0.11 | 0.33 | -0.08 | 0.26 | 0.04 | 0.10 |
|  | IgM | 0.17 | 54.69 | -- | 1.40 | 8.59 | 2.74 | 3.54 | -- | 0.22 | 2.41 |
|  | IgG1 | -0.17 | 0.22 | 0.26 | -0.02 | 0.05 | 0.34 | -0.21 | 0.21 | -0.25 | -0.06 |
|  | IgG2 | -0.19 | 0.23 | 0.49 | -0.02 | 0.03 | 0.33 | -0.16 | 0.17 | 0.03 | 0.04 |
|  | IgG3 | -0.10 | 0.01 | 0.26 | -0.02 | 0.02 | 0.25 | -0.27 | 0.51 | 0.27 | -0.02 |
|  | IgG4 | -0.21 | 0.18 | 0.99 | 0.11 | 0.06 | 0.46 | 0.14 | -0.02 | -0.05 | 0.12 |
| Controls | APOA1 | -0.28 | 0.38 | 0.71 | 0.19 | 0.15 | 0.57 | 0.18 | 0.47 | 0.29 | 0.25 |
|  | SCCA–1 | 0.20 | 0.12 | 0.58 | 0.00 | -0.11 | 0.17 | -0.23 | 0.05 | 0.11 | -0.09 |
|  | Albumin | -0.09 | 0.32 | 0.30 | 0.12 | -0.03 | 0.40 | -0.21 | 0.20 | -0.03 | 0.07 |
|  | Involucrin | -0.19 | 0.26 | 0.54 | 0.06 | 0.12 | 0.41 | 0.13 | 0.42 | 0.45 | 0.07 |
|  | Total protein | 0.02 | 0.12 | 0.03 | -0.02 | 0.05 | 0.13 | -0.04 | -0.05 | -0.10 | -0.01 |

**1.** Associations with analytes with ≥85% LLOQ modelled using random effects linear regression; coefficients represent change from reference group in log analyte concentration. Associations with analytes with <85% LLOQ modelled using random effects logistic regression; coefficients represent odds ratios for detectable analyte concentration compared with reference group. **2.** Adjusted for age and haemoglobin only. **3**. Linear trend for change in log analyte concentration (if ≥85% LLOQ)/change in odds of analyte detection (if <85% LLOQ) with one unit increase in exposure category. **4**. PSA categories: None, low positive (<4 ng/mL), high positive (≥4 ng/mL). **5.** Vaginal pH categories 3.6-4.1; 4.4-4.7; and 5.0 and above. **6.** Neutrophil categories: No cells, 1–10 cells, 11–50 cells, >50 cells. **7.** Analytes with <85% observation LLOQ.

| Colour legend: | p≥0.1 |  | p<0.10 |  | p<0.05 |  | P<0.01 |  | P<0.005 |  | P<0.001 |  |
| --- | --- | --- | --- | --- | --- | --- | --- | --- | --- | --- | --- | --- |
