# Supplementary material for: Immune Activation in the Female Genital Tract: Expression Profiles of Soluble Proteins in Women at High Risk for HIV Infection
Source: PLoS One. 2016 Jan 27;11(1):e0143109. doi: 10.1371/journal.pone.0143109 (PMC4729472; doi:10.1371/journal.pone.0143109)
Supplement: S1 Text — (DOCX) [file pone.0143109.s003.docx]

**S1 Text. Normalisation to account for dilution factor in the cervicovaginal lavage**

The primary advantage of using cervicovaginal lavage is that it can recover all material present in the lower genital tract; however, one drawback is the altered concentration of analytes due to an unknown dilution factor *(1)*. Protein normalization can be used to adjust for relative concentration differences, but since protein may also be correlated with variation, e.g. reproductive tract infections, we choose to also investigate normalization with involucrin, a structural protein expressed in the stratifying squamous epithelia, including vaginal epithelium *(2)*. To assess for variability of the effect of dilution we compared the intra-class correlation coefficient (ICC) of each log-transformed raw, protein- and involucrin-normalized biomarker. We found there was more total variation (larger standard deviation) in the involucrin-normalized biomarkers than in raw or protein-normalized biomarkers. There was also more absolute within-woman variation (higher σw) with involucrin-normalized data. However, in general, the ICC was higher with the involucrin-normalized data than with the raw or protein-normalized data. Therefore, in the involucrin-normalized data, although there was more absolute variation in the data, observations from the same woman become relatively more constant, and between-women differences made up a larger proportion of the total variation. Generally, the ICC was lowest with protein-normalized data, indicating that observations from the same woman were relatively less constant, and within-women differences made up a relatively larger proportion of the total variation (Table S6). Based on these findings and our concerns about protein correlations with background variation, we analyzed the data using the raw data only.

References

1. C. S. Dezzutti, C. W. Hendrix, J. M. Marrazzo, Z. Pan, L. Wang, N. Louissaint, S. Kalyoussef, N. M. Torres, F. Hladik, U. Parikh, J. Mellors, S. L. Hillier, B. C. Herold, K. A. Kelly, Ed. Performance of swabs, lavage, and diluents to quantify biomarkers of female genital tract soluble mucosal mediators., *PLoS One* **6**, e23136 (2011).

2. M. H. Dinh, E. a Okocha, A. Koons, R. S. Veazey, T. J. Hope, Expression of structural proteins in human female and male genital epithelia and implications for sexually transmitted infections., *Biol. Reprod.* **86**, 32 (2012).
